# Developmental differences in the prospective organisation of goal‐directed movement between children with autism and typically developing children: A smart tablet serious game study

**DOI:** 10.1111/desc.13195

**Published:** 2021-12-06

**Authors:** Yu Wei Chua, Szu‐Ching Lu, Anna Anzulewicz, Krzystof Sobota, Christos Tachtatzis, Ivan Andonovic, Philip Rowe, Jonathan Delafield‐Butt

**Affiliations:** ^1^ Laboratory for Innovation in Autism University of Strathclyde Glasgow Scotland UK; ^2^ Faculty of Humanities and Social Sciences University of Strathclyde Glasgow Scotland UK; ^3^ Faculty of Psychology University of Warsaw Warsaw Poland; ^4^ Department of Electronic and Electrical Engineering University of Strathclyde Glasgow Scotland UK; ^5^ Department of Biomedical Engineering University of Strathclyde Glasgow Scotland UK

**Keywords:** autism, embodiment, feed‐forward and feed‐back mechanisms, prospective motor control, smart tablet serious game paradigms

## Abstract

Movement is prospective. It structures self‐generated engagement with objects and social partners and is fundamental to children's learning and development. In autistic children, previous reports of differences in movement kinematics compared to neurotypical peers suggest that its prospective organisation might be disrupted. Here, we employed a smart tablet serious game paradigm to assess differences in the feedforward and feedback mechanisms of prospective action organisation, between autistic and neurotypical preschool children. We analysed 3926 goal‐directed finger movements made during smart‐tablet ecological gameplay, from 28 children with Childhood Autism (ICD‐10; ASD) and 43 neurotypical children (TD), aged 3–6 years old. Using linear and generalised linear mixed‐effect models, we found the ASD group executed movements with longer movement time (MT) and time to peak velocity (TTPV), lower peak velocity (PV), with PV less likely to occur in the first movement unit (MU) and with a greater number of movement units after peak velocity (MU‐APV). Interestingly, compared to the TD group, the ASD group showed smaller increases in PV, TTPV and MT with an increase in age (ASD × age interaction), together with a smaller reduction in MU‐APV and an increase in MU‐APV at shorter target distances (ASD × Dist interaction). Our results are the first to highlight different developmental trends in anticipatory feedforward and compensatory feedback mechanisms of control, contributing to differences in movement kinematics observed between autistic and neurotypical children. These findings point to differences in integration of prospective perceptuomotor information, with implications for embodied cognition and learning from self‐generated action in autism.

## 1 INTRODUCTION

Children move to engage the world of people and objects, and to learn from those experiences (Delafield‐Butt, 2018; Reed, 1996; Trevarthen & Delafield‐Butt, 2017). They test the world with action and learn its responses (Baldwin, 1895; Piaget, 1953). From the infant's first simple movements (banging, sucking, smiling) to the serially organised complex projects of young children (grasping, stacking, climbing, playing), self‐generated movement forms the bedrock of psychological experience on which learning, cognition and social understanding develop (Clark, 1999; Koziol et al., 2012; Wilson, 2002; Delafield‐Butt, 2018; Pezzulo et al., 2008; Pezzulo & Castelfranchi, 2009; Trevarthen & Delafield‐Butt, 2017).

Efficient prospective control of actions, processes involved in predicting, anticipating and achieving goals in the near or distant future (von Hofsten, 1993), is crucial to the structure of sensorimotor experiences (Delafield‐Butt & Gangopadhyay, 2013). Each action must be guided with an anticipation of its future effect (Bernstein, 1967; Trevarthen, 1984) as it moves experience from ‘where one is’ to ‘where one wants to be’ as they bring the person usefully forward in time (von Hofsten, 1993, 2007; Lee, 2009). Movement, and the motor system on which it depends, enables development of a ‘sensorimotor intelligence’ that underpins all experience, learning and social interactions (Delafield‐Butt & Trevarthen, 2015; Trevarthen & Delafield‐Butt, 2015; Piaget, 1953; 1954). This early, self‐generated learning is evident in the fine detail of movement from birth (Delafield‐Butt & Gangopadhyay, 2013), and high‐precision analysis of its particular motor form can indicate developmental risk (Delafield‐Butt et al., 2018; Craig et al., 2000; Torres et al., 2016). Disruption to movement in early childhood can thwart learning. Early childhood motor delays or difficulties are predictive of later socio‐communicative difficulties (MacDonald et al., 2014) and can be the first sign of neurodevelopmental disorder (Gillberg, 2010).

Recent evidence of a subtle, but significant motor disruption associated with autism spectrum disorder (hereafter, autism or ASD) has led to a growing body of research on sensorimotor difficulties and differences in autism at the kinematic, action and behavioural levels, from impairments in motor coordination (Fournier et al., 2010) and motor planning (Gowen & Hamilton, 2013), to differences in action imitation (Williams et al., 2004) and its affective expression (Casartelli et al., 2020). Movement differences have implications on how we understand socio‐communicative development in autistic individuals (Bhat et al., 2011) and how they make sense of the world in shared engagement with others (Trevarthen & Delafield‐Butt, 2013).

Notably, not all movement differences are deficits per se. Individuals optimise movement kinematics in reaction to external task constraints that affect the efficiency and accuracy of their movements in relation to a goal (Elliott et al., 2020)—a fundamental principle of prospective action organisation (Lee, 2009). While it has been suggested that there may be some form of neuromotor disruption in autism, such as greater motor noise or imprecise muscular timing, these can be understood as internal constraints on goal achievement. In this way, kinematic differences in autism can be seen as an adaptive developmental response to optimise movement to achieve goals in the presence of intrinsic neuromotor constraints. This is supported by recent empirical work reviewed by Elliott and colleagues (2020) showing that motor learning in autistic individuals leads to different kinematic patterns (such as increased spatial variability). Importantly, motor control and learning nevertheless appear intact as autistic individuals successfully solved the motor task. Interestingly, they differed from neurotypical controls on how they relied on visual (Hayes et al., 2018) and contextual (Foster et al., 2020a) information during motor learning. This suggests that visuomotor integration plays a different role in learning through motor experiences in this adaptive developmental system.

In this paper, we advance a multiple‐process model of goal‐directed aiming following a comprehensive framework for analysis of goal‐directed movement kinematics (Elliott et al., 2010, 2017). Movement kinematics are directly related to the neuro‐ and psycho‐motor processes underlying movement generation, including perception, planning, feedforward and feedback control (Bootsma et al., 2004; Fitts, 1954; Kawato, 1999; MacKenzie et al., 1987; Wolpert et al., 1995; Wolpert & Ghahramani, 2000; Woodworth, 1899; Lee, 2009).

**RESEARCH HIGHLIGHTS**

- Differences in movement kinematics have been increasingly reported in autism spectrum disorder, and highlighted as a potential contributing factor to its social features.
- We analysed close to four thousand goal‐directed swipes from preschool and autistic children, assessed ecologically during smart‐tablet gameplay.
- We found group differences in the anticipatory feedforward and compensatory feedback components of children's goal‐directed actions, arising from different trends with increasing age.
- Our findings reveal developmental differences in the prospective organisation of movement, which is fundamental to children's learning through motor experience.

Kinematics variables describe the movement and reflect its motor plan. For example, ‘peak velocity’ (PV), ‘time to peak velocity’ (TTPV) and ‘PV of the first MU’ reflects the execution of an efficient goal‐directed movement using feedforward control, and kinematics such as the ‘percent time after PV’ and the number of MUs reflects the recruitment of feedback control, while overall movement time (MT) reflects the speed‐accuracy trade‐off in generating efficient and accurate goal‐directed movements (Elliott et al., 2010, 2017).

### 1.1 Movement kinematics in autism

Compared to neurotypical controls, atypical movement kinematics have been reported in autism in a variety of tasks, including longer MT s (Campione et al., 2016; Forti et al., 2011; Glazebrook et al., 2006, 2009; Mari et al., 2003; Stoit et al., 2013; Yang et al., 2014) as well as lower peak velocities (Forti et al., 2011; Glazebrook et al., 2006; Mari et al., 2003) and longer times to PV (Campione et al., 2016; Glazebrook et al., 2006, 2009), all of which point to differences in feedforward control. However, some studies did not find evidence of group differences in MT s (Dowd et al., 2012; Fabbri‐Destro et al., 2009; Papadopoulos et al., 2012) or peak velocities (Campione et al., 2016; Dowd et al., 2012; Yang et al., 2014). One study has suggested, to the contrary, that autistic individuals execute movements with greater PV than neurotypical individuals (Cook et al., 2013). In addition, peak acceleration, also thought to be associated with feedforward control (Elliott et al., 2010), was lower in autistic young adults compared to neurotypical controls (Glazebrook et al., 2006). However, peak acceleration has not been widely studied and group differences were not found in children's reaching movements (Campione et al., 2016) or simple point‐to‐point movements (Dowd et al., 2012).

Few studies have investigated differences in kinematics related to feedback control and the direction of differences between ASD and neurotypical (TD) populations remain unclear. Three studies investigated the relative duration of the deceleration phase, quantified as the percentage of MT after PV occurred, and did not find differences (Campione et al., 2016; Glazebrook et al., 2006; Rinehart et al., 2006). However, there is some evidence that autistic individuals may require a greater extent of feedback processing to control movement, as their movements may be jerkier (Cook et al., 2013; Yang et al., 2014) and comprise more MUs (Forti et al., 2011; Yang et al., 2014).

A gap in the literature is in the consideration of developmental changes in movement kinematics. First, differences between ASD and TD populations in how the kinematic organisation of movement develops can obscure group differences, or change the direction of effects observed at different ages. Second, in children, motor skills are still maturing and can develop significantly across the span of months. Earlier investigations of movement kinematics studied children of different ages, matching groups for age during sampling (Campione et al., 2016; Dowd et al., 2012; Forti et al., 2011; Mari et al., 2003; Rinehart et al., 2006) or including it as a covariate in the analysis (Dowd et al., 2012). However, including a mix of ages in the study design as large as a 5‐year range in Dowd and colleagues’ (2012) study can introduce substantial within‐group variability on top of within‐individual movement variability inherent to the motor system and particularly when motor skills are developing. This means that in these earlier studies, differences between ASD and TD groups may have been confounded or obscured in the presence of variability due to age, in their relatively small study samples.

More importantly, developmental changes in the kinematic structure of movement provide insight into the development of goal‐directed movements. Like in adults, infant reaches are structured into phases of acceleration and deceleration or ‘MUs’, including a dominant MU covering the most distance to the target – the primary transport unit (von Hofsten, 1991). With development, the number of MUs decreases, the PV or primary transport unit occurs earlier and covers an increasing proportion of the target distance (Berthier & Keen, 2006; Konczak et al., 1997, 1995; Newman et al., 2001; von Hofsten, 1991). Reach trajectories become straighter (Berthier & Keen, 2006; von Hofsten, 1991). By the end of 2 years, adult‐like movements with a single bell‐shaped velocity profile start to be produced predominantly (Berthier & Keen, 2006; Konczak & Dichgans, 1997) but the quality of reaches continue to improve throughout childhood, including reduced variability in reach endpoint (Contreras‐Vidal, 2006; King et al., 2012). This body of research, conducted in neurotypical populations, suggest that with development, there is a reduced reliance on later corrective feedback movements, as the initial planning phase becomes more efficient (Deutsch & Newell, 2005), potentially through the development of more accurate motor plans. If this developmental process is altered in autism, this could indicate that differences in motor planning or execution can have downstream effects on motor control processes recruited for producing efficient goal‐directed movement.

Tablet‐based technology has become more widely available as accessible research tools, and used to study movement kinematics in autistic children (Dowd et al., 2012; Papadopoulos et al., 2012; Rinehart et al., 2006), but the developmental significance of the movements studied using these tools is often overlooked. Specifically, movements made on a tablet surface are usually part of a two‐step movement: first to bring the finger or pen to the tablet surface, before making the desired movement within the tablet environment. Research using new technology and smart‐tablet technology should consider that devices do not just provide a virtual environment within their workspace, but are also objects situated in the real‐world environment.

### 1.2 Current study

In summary, theoretical advances from an embodied cognition framework highlight the role of early sensorimotor differences in socio‐communicative development through learning. Movement kinematics provide a window into the processes involved in the control of movement and differences at this level have been reported in autistic compared to neurotypical individuals. Differences in developmental trends may indicate if motor control processes are recruited to different extents with development. Smart‐tablet technology has provided easy access to the recording of movement kinematics, but little consideration has been given to the developmental significance of such movements.

In this study, kinematic analysis was conducted on goal‐directed movements made by 3‐ to 6‐year‐old children during smart‐tablet gameplay, involving moving food pieces within a start area onto plates within an end area (Anzulewicz et al., 2016). The food‐to‐plate movements are considered to be conceptually equivalent to the second step of a two‐step movement where the target distance from food‐to‐plate modulates the difficulty of the movement, preceded by a movement to bring the finger onto the food area of the touch screen. Two‐step actions such as a reach‐to‐place task have been used to investigate prospective control (Gottwald et al., 2017) and kinematics of the second step were sensitive to changes in task demands, relating to differences in planning and prospective control of the second movement step (Gottwald, 2018).

We approached the investigation of differences between ASD and TD groups in the kinematic organisation of movement in two steps. First, we explored the validity of kinematic variables proposed in the multiple process model, as indicators of feedforward and feedback control in the context of smart‐tablet gameplay. PV, PV of the first MU (PV1), and TTPV were selected a priori as kinematic variables related to feedforward control; MUs, percent time after PV (i.e. the deceleration phase, %Dec) related to feedback control; and MT related to both feedforward and feedback control. Movement units after peak velocity (MU‐APV) and a binary variable, whether peak velocity was found in the first MU (PV1‐b), were further explored as potential indicators of feedback and feedforward control, respectively. Next, we investigated the relationship between ASD diagnosis, target distance and age on selected movement kinematic variables. We hypothesised that (1) autistic children will differ from neurotypical children in the extent of both feedforward and feedback control (effect of ASD); (2) in how kinematics relating to the feedforward and feedback processes develop with age (interaction effect of ASD × age) and (3) how they alter kinematics in relation to target distance, an indicator of goal difficulty (Interaction effect of ASD × Dist).

## 2 METHODS

### 2.1 Sample

Data from Anzulewicz and colleagues (2016) on finger movements during smart‐tablet (Apple Inc., iPad mini 2 2013, iOS version 7.0) gameplay of the ‘Sharing’ game were analysed. The dataset consists of 82 children aged between 3 and 6 years old, including 37 children had a clinical diagnosis of ICD‐10 Childhood Autism (ASD) (12 female) and 45 typically developing (TD) children (13 female). Children in the ASD group were recruited from specialist therapeutic centres in Krakow, Poland, where they were presenting for professional input for symptoms related to autism. The majority (*n* = 33) did not have any comorbid disorders, four participants were considered co‐morbid with ‘intellectual impairment’ without sensory or motor deficits, two were diagnosed with Asperger's Syndrome, and one was considered ‘high functioning’. The ‘Sharing’ game involved moving food pieces presented in a central area towards one of the four plates in the game scene. Participants were given 2 min of practice to familiarise with the task before 5 min of data collection during gameplay. Kinematic data were sampled at a spatial resolution of 2048 × 1536 pixels at 326 pixels per inch, and a temporal resolution of 60 Hz, as determined by the smart‐tablet device characteristics. Further description of the dataset can be found in the report by Anzulewicz and colleagues (2016). This study conforms to the ethical principles set out in the Declaration of Helsinki and was approved by the University of Strathclyde Ethics Committee. Informed consent for children's participation in the study was provided by their parents. Data had been anonymised prior to access by the first author for the present investigation. Apart from participant age and gender, no other personal information was linked to the touchscreen movement data.

**FIGURE 1:**
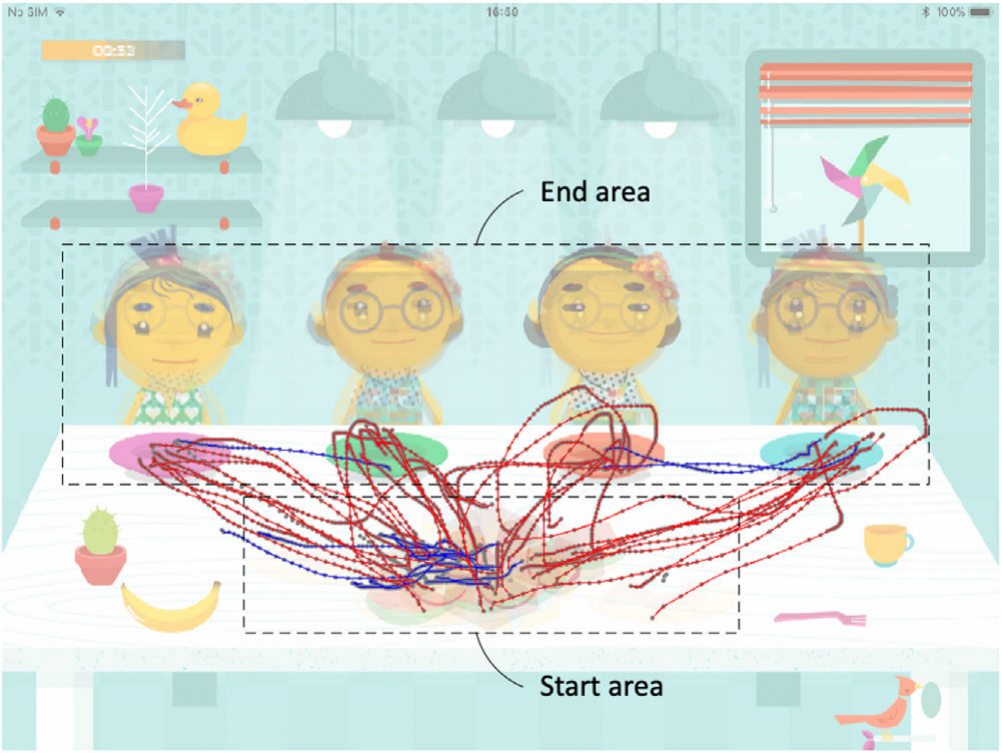
**‘**Sharing’ game, showing food‐to‐plate swipes from one participant. Participants made swipes from food presented in different locations within the food area, to locations within the end area. Participants predominantly ended their movement in the plate areas, but the game mechanics regarded a ‘successful’ swipe as one that moved the food to a plate or to the location of any cartoon characters. This figure shows examples of the successful food‐to‐plate swipes (red) and unsuccessful swipes (blue) excluded from the analysis in this study

Goal‐directed finger swipes were included for analysis in this study. Unlike paradigms on point‐to‐point movements, the ‘Sharing’ game did not have specific start and end‐points; therefore goal‐directed swipes were defined as swipes beginning in the food area and ending in the target area (food‐to‐plate swipes, see Figure 1). We excluded swipes not suitable for kinematic analysis: swipes made with multiple touches (where more than one gesture was registered at the same time) were excluded as a unique swipe path could not be distinguished; and swipes consisting of less than five data points were excluded to permit velocity derivatives using a five‐point stencil (see Procedure).

To increase the validity of our analysis to goal‐directed swipes, we further restricted the analysis to swipes likely to be performed according to the task‐demands. We excluded: first, food‐to‐plate swipes from participants who made at least 10% of food‐to‐plate swipes out of the total swipes made during gameplay; second, outliers of food‐to‐plate swipes based on MT (>2.0 s) and target distance (>70 mm) as these are unlikely to be swipes aimed at reaching a single goal location efficiently; finally, swipes with a straightness index (ratio of distance moved to target distance) greater than 1.5. This criteria for straightness index was selected as it excluded most of the outliers based on visual‐inspection of a box‐plot, and was guided by reports that straightness ratio of reaching movements decrease to about 1.4 by 3 years of age (Berthier & Keen, 2006).

### 2.2 Kinematic variables (a priori)

*MT* was defined as the time from touch begun to touch end.

*MU* was a count variable defined as a velocity maximum comprising an acceleration and deceleration phase cumulatively resulting in a velocity change of 8 mm/s or more. Velocity maxima were included only if they were greater than 5% of PV. Swipes were visually inspected to ascertain that this criterion excluded small changes in velocity in the count of MUs (Achermann et al., 2020; von Hofsten, 1991). The start of the first MU was defined as the acceleration phase where velocity increases from the first velocity minimum or from the time touch was detected, to a velocity maximum. The end of the last MU was defined as the deceleration phase, where velocity decreases from a velocity maximum to the last velocity minimum, or the touch was detected to end.

*PV* was the value of the greatest magnitude of velocity resulting from the movement.
*PV of the first MU (PV1)* was the value of the maximum velocity of the first MU.
*TTPV* was the time from touch begun to the time PV was reached.
*Deceleration phase (%Dec)* was the ratio of time after PV to movement end, expressed as a percentage.

### 2.3 Kinematic variables (exploratory analyses)

*MU‐APV* was the number of MUs occurring after PV.

*PV of the first MU (PV1‐b)* was a binary variable, whether PV1 was found in the first MU.

### 2.4 Predictors

ASD diagnosis (ASD), defined as clinical diagnosis of ICD‐10 Childhood Autism, and participant age (age), measured in months, were included as predictors of kinematic variable at the cluster level.

Target distance (Dist), defined as the displacement between the start and end position of the swipe, was included as a predictor of the kinematic variable at the swipe level. This was an ordered categorical variable in 10‐mm intervals.

### 2.5 Data preprocessing

Data consisting of timestamps and positional coordinates recorded in Apple Developer's UITouch object were pre‐processed in Python 3.7. Movement start was defined as the time when a touch was detected (UITouch = 0). Movement end was defined as the time an ongoing touch was detected to end (UITouch = 3). Invalidly recorded swipes, without a touch detected, moved (UITouch = 1), and end structure, were excluded from analysis.

Movement *x*‐ and *y*‐position vectors were filtered using a fourth‐order, zero‐phase shift, 8‐Hz low pass Butterworth filter (Bartlett, 2007) (see Supplementary Material for more information on how this filter frequency was selected). Finally, velocity magnitude was calculated as the vector sum of *x*‐ and *y*‐velocity vectors and kinematic outcome variables were calculated for each swipe according to the definitions described.

### 2.6 Data analysis

Data analysis was conducted in R (version 3.6) and RStudio (version 1.2). Mixed effect models were fitted using the lme4 (Bates et al., 2015) and glmmTMB packages (Brooks et al., 2017).

A chi‐squared test was conducted to test if there was a group difference in the number of swipes excluded from analysis. Descriptive statistics by group were obtained for age (mean, standard deviation and range) and sex (frequency and proportion). Numbers of swipes made for each category of Dist were cross‐tabulated by group. Group differences in means and standard deviations of participant age were tested using a *T*‐test, differences in distribution of sex using a chi‐squared test and differences in median Dist category using a Mann–Whitney test. The distribution of each swipe kinematic outcome by group was inspected using violin plots for continuous or count variables, and a barplot for the binary variable PV1‐b.

To account for the nesting of swipe data by individual, linear and generalised linear mixed models were fitted for each swipe kinematic outcome with Dist centred to the median category (30–40 mm), and age was scaled to years and mean‐centred (4.7 years). Linear regression models were fitted for five outcome variables: PV1, %Dec; and log‐transformed variables MT, TTPV and PV. General linear regression models (zero‐truncated Poisson log‐link models) were fitted for MU. As exploratory analyses, following model diagnostics for the a priori kinematic variables, we fitted generalised linear mixed models for kinematic variables PV1‐b (logistic regression) and MU‐APV (Poisson log‐link regression). Finally, we computed pairwise correlations between all the kinematic outcomes considered in the study to strengthen the interpretation of the movement kinematic as indicators of feedforward and feedback control.

### 2.7 Model building and diagnostics

The top‐down model building procedure recommended by Zuur and colleagues (2009) was followed. We considered random intercepts to account for the non‐independence in swipes made by the same subject as part of our experimental design, but additionally included random slopes for all kinematic variables as improved the models (Supplemental Table 4). For multivariate conditional models, ASD, Dist and age were included as hypothesised fixed effects, but we only included interaction effects in the final model if they improved the model to be able to estimate relevant parameters more accurately and precisely to answer the experimental questions. As part of experimental hypotheses, we tested for the effect of ASD × Dist and ASD × age. We also considered an interaction effect of Dist × age to control for age effects on the slope of Dist. No interaction effect of Dist × age was found for all models except for PV1 (Supplemental Table 5). As the distribution of PV, TTPV and MT was positively skewed (Figure 4) and residuals obtained from modelling each of these variables were not normally distributed, we log‐transformed PV, TTPV and MT and re‐fitted the models using the same model building procedure. Final models were fitted using the REML estimator for linear models and ML estimator for general linear models. Parameter estimates with 95% confidence intervals and *p* values were obtained by applying Type III ANOVA Satterthwaite and Wald's approximation to degrees of freedom for linear and general linear models, respectively. Further information on the model building procedure is provided in Supplementary Material.

**FIGURE 2:**
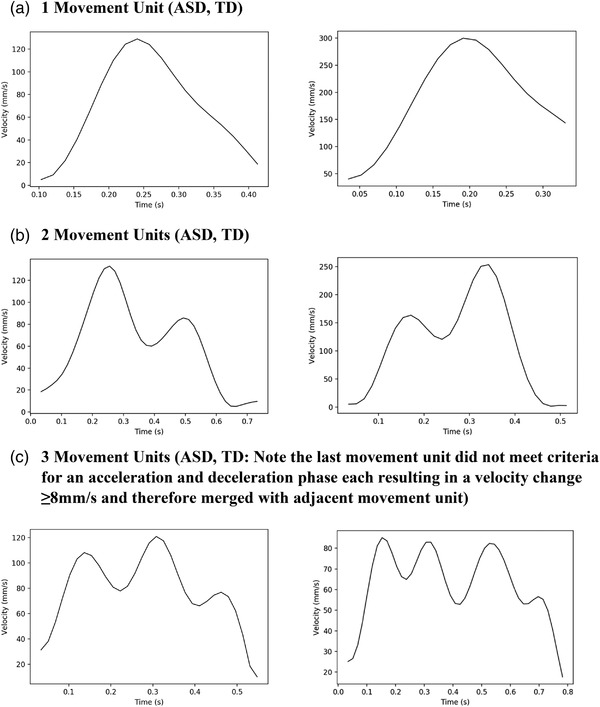
Swipe velocity profiles. Each participant may execute a mix of velocity profiles, representative examples for ASD (left) and TD (right) children are given for swipes with (a) 1, (b) 2, or (c) 3 movement units

### 2.8 Exploratory kinematic variables

#### 2.8.1 MUs after peak velocity (MU‐APV)

As fixed effects explained only 1% of the variance in %Dec, we sought to identify another kinematic variable indicative of feedback processing, that was less susceptible to variability in whether the PV occurred in the first MU. We derived a count variable, MU‐APV.

#### 2.8.2 PV of the first MU (PV1‐b)

Due to heteroscedasticity in residual variance found in the model fit for PV1 and because the swipe movement profile varied in whether the largest peak in velocity occurred in the first MU (Figure 2), we derived a binary variable indicating whether PV was found in the first MU (PV1‐b).

### 2.9 Sensitivity analyses

We reran the final models (for MT, TTPV, PV, PV1‐b and MU‐APV only) on a stricter dataset, which further excluded swipes that did not meet the criteria of <5‐mm distance covered before first minima and <5‐mm distance covered after last minima, if applicable. This was to exclude: (1) movements that decelerate over a significant proportion of the target distance upon contacting the touchscreen surface before making the food‐plate swipe, which would invalidate the Dist category; and (2) movements resulting from a strategy to slowly reduce the distance to the goal, before quickly accelerating towards goal while lifting the finger off the touchscreen surface, which do not have the same kinematic form as accurate goal‐directed movements even if they achieved the task‐demand in the gameplay context.

### 2.10 Data availability

Derived and analysed kinematic data, Python scripts used to generate kinematic data from raw touchscreen data and R scripts used to generate kinematic data analysis are available on https://osf.io/xjdf8/.

## 3 RESULTS

### 3.1 Analysis sample

A total of 4917 food‐to‐plate swipes were made by 82 participants. Among these, 159 (3.2%) swipes were not suitable for the present analysis as they resulted from ‘multiple touch’ where more than one swipe was registered at the same time (*n* = 118, 2.4%), containing fewer than five data points (*n* = 7, 0.1%), or resulted in swipe distances shorter than the shortest food‐plate distance (*n* = 34, 0.7%). After exclusion, 3926 swipes from 71 participants (43 TD, 28 ASD), formed our sample of goal‐directed food‐to‐plate swipes. 2593 swipes (66.0%) were made by TD participants and 1333 swipes (34.0%) were made by ASD participants. ASD diagnosis did not influence whether swipes were more likely to be excluded (χ^2^ (1) = 0.385, *p* value = 0.535). See Supplemental Table 1 for a breakdown of the excluded swipes.

The mean number of goal‐directed swipes made per individual was 66 swipes, and on average this was 20 swipes greater for TD participants (73 swipes) than ASD participants (55 swipes) (*T*(69) = 2.99, *p* = 0.004).

**FIGURE 3:**
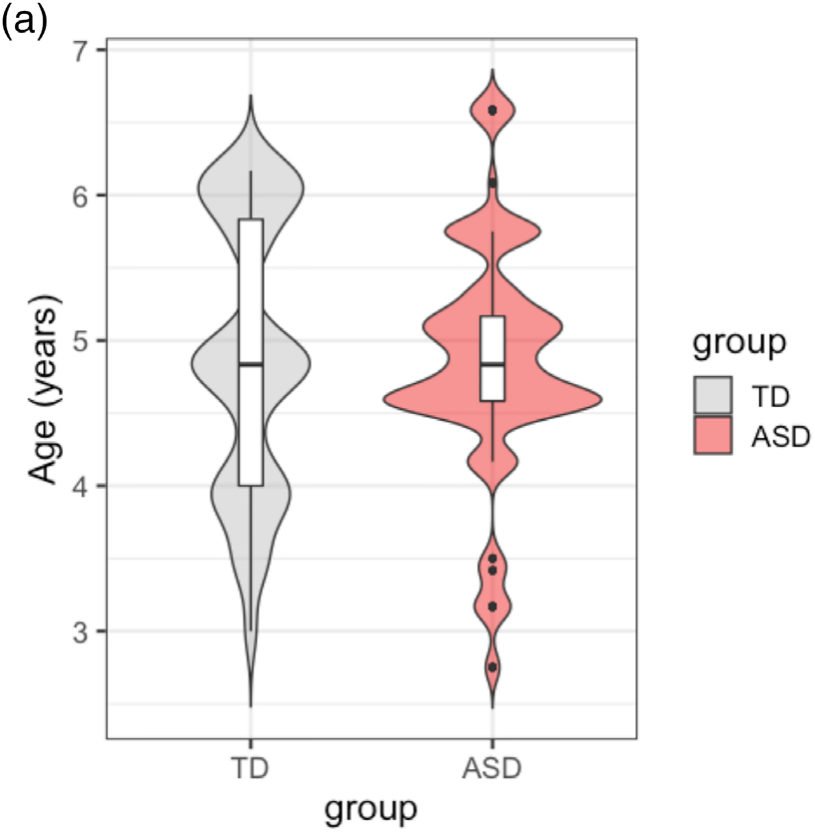
Violin plot showing distribution of participant age by ASD diagnosis (Black: TD, Red: ASD)

Forty‐seven (66.2%) participants in the analysis sample were male, and the proportion of male and female did not differ between ASD and TD groups (χ^2^ (1) = 0.621, *p* = 0.431). Participants’ age ranged from 2.8 to 6.6 years, with a mean of 4.7 years (s.d = 0.905). Means and variance of age were not different between ASD and TD groups (*T*(69) = −0.184, *p* = 0.855; *F*(42,27) = 1.10, *p* = 0.801). Swipes made by participants in the TD and ASD group had displacement ranging from 13.5 to 70.0 mm, leading to six ordered categories of 10‐mm intervals from 11  to 70 mm. Proportions in respective categories of Dist were marginally significantly different between TD and ASD groups (χ^2^ (5) = 11.2, *p* = 0.047). Proportionally, children in the ASD group performed marginally more swipes in the 30–40‐mm category and marginally less swipes in the 10–20 and 20–30‐mm category. The median Dist was 30–40 mm. See Tables 1, 2, 3 and Figure 3 for details.

**TABLE 1:** Swipes excluded and analysed

Food‐to‐plate swipes	Total (*N* = 4917)	TD (*N* = 3233)	ASD (*N* = 1684)	*p* value
Swipes excluded	991 (20.2%)	640 (19.8%)	351 (20.8%)	0.406
Analysis sample	3926 (79.8%)	2593 (80.2%)	1333 (79.2%)	

**TABLE 2:** Analytic sample: participant age and swipes per individual, by ASD diagnosis

Participants	Total (*N* = 71)	TD (*N* = 43)	ASD (*N* = 28)	*p* value
Sex				
*Male, N (%)*	47 (66.2%)	30 (69.8%)	17 (60.7%)	0.431
*Female, N (%)*	24 (33.8%)	13 (30.2%)	11 (39.3%)	
Age, years				
*Mean (sd)*	4.7 (0.9)	4.7 (0.9)	4.7 (0.9)	0.855
*Range*	2.8–6.6	3.0–6.2	2.8–6.6	n.a
Swipes per participant				
*Mean (sd)*	66.0 (27.0)	73.3 (25.6)	54.8 (25.4)	0.004
*Range*	11–145	18–145	11–96	n.a

Abbreviation: TD, typically developing.

**TABLE 3:** Analytic sample: characteristics of goal‐directed swipes by ASD diagnosis

Swipes	Total *n* (%)	TD *n* (%)	ASD *n* (%)	*p* value
Total	3926 (100%)	2593 (66.0%)	1333 (34.0%)	n.a
Target distance				
10–20 mm	232 (5.9%)	172 (6.6%)	60 (4.5%)	0.0468
20–30 mm	1207 (30.7%)	808 (31.2%)	399 (29.9%)	
30–40 mm	743 (18.9%)	467 (18.0%)	276 (20.7%)	
40‐–50 mm	851 (21.7%)	552 (21.3%)	299 (22.4%)	
50–60 mm	643 (16.4%)	429 (16.5%)	214 (16.1%)	
60–70 mm	250 (6.4%)	165 (6.4%)	85 (6.4%)	

Abbreviation: TD, typically developing.

### 3.2 Linear and generalised‐linear mixed effect models

In this section, we report on the final linear and generalised‐linear mixed effect models for log‐transformed variables MT, PV, TTPV, and variables PV1‐b and MU‐APV (Table 4, fixed effect; Table 5, random effects). Assumptions of normality of residuals, homogeneity of variance and linearity were met following log‐transformation (Supplemental Figure 1) and MU‐APV was not overdispersed (dispersion ratio = 0.902). The total variance explained ranged from 26.9% to 73.6%, with fixed effects explaining 12.5%–35.3% of the total variance (Table 5). Exponentiated (multiplicative) coefficients are reported for log‐transformed variables MT, TTPV, PV, odds ratios (OR) for PV1‐b and incidence rate ratios (IRR) for MU‐APV (Table 4). Predicted marginal effects of ASD, age and Dist are shown in Figures 5 and 6.

**TABLE 4:** Final models. Linear, logistic and Poisson mixed effect models. Fixed effects with exponentiated coefficients for movement time, peak velocity, time to peak velocity; odds ratios for peak velocity 1MU‐b and incidence rate ratios for movement units APV. Fixed effects were calculated at the median Dist category (30–40 mm) across all swipes and mean age (4.7 years) across all participants

	MT (s)	PV (mm/s)	TTPV (s)	PV1‐b	MU‐APV
***Fixed effects***	*Coefficient (95% CI)*	*Coefficient 95% CI)*	*Coefficient (95% CI)*	*Odds ratios 95% CI)*	*Incidence rate Ratios 95% CI)*
Intercept	0.70*** (0.63–0.77)	114.11*** (105.96–122.88)	0.28*** (0.25–0.32)	4.45*** (3.43–5.78)	0.29*** (0.24–0.35)
Dist	1.10*** (1.09–1.12)	1.17*** (1.15–1.18)	1.13*** (1.11–1.15)	0.75*** (0.70–0.81)	1.33*** (1.26–1.41)
Age	0.71*** (0.64–0.79)	1.25*** (1.16–1.35)	0.74*** (0.65–0.83)	1.89*** (1.52–2.35)	0.61*** (0.51–0.72)
ASD	1.13 (0.96–1.33)	0.92 (0.82–1.03)	1.20* (1.00–1.44)	0.56** (0.38–0.82)	1.36* (1.01–1.83)
ASD × age	1.33** (1.11–1.60)	0.88 (0.77–1.00)	1.21 (0.99–1.48)	n.a	1.35* (1.02–1.80)
ASD × Dist	n.a	n.a	n.a	n.a	0.91* (0.84–0.99)

Abbreviations: MT, movement time; MU‐APV, movement units after peak velocity; PV, peak velocity; PV1‐b, peak velocity of the first movement unit; TTPV, time to peak velocity.

* *p *< 0.05.

** *p *< 0.01.

*** *p *< 0.001.

**TABLE 5:** Final models. linear, logistic and Poisson mixed effect models. Random effects coefficients for residuals, intercept, slope, and correlation between intercept and slope, marginal/conditional R2; intra‐class correlation, deviance statistic and AIC criteria

	MT (s)	PV (mm/s)	TTPV (s)	PV1‐b	MU‐APV
***Random effects***	*Estimates*	*Estimates*	*Estimates*	*Estimates*	*Estimates*
σ^2^	0.08	0.09	0.21	3.29	1.17
τ_00 Subject_	0.13	0.06	0.14	0.62	0.30
τ_11 Subject. Dist_	0.00	0.00	0.00	0.03	0.01
ρ_01 Subject_	−0.48	−0.32	−0.11	−0.52	−0.86
ICC	0.61	0.41	0.40	0.16	0.20
Marginal *R* ^2^/conditional *R* ^2^	0.296/0.729	0.350/0.614	0.201/0.517	0.123/0.263	0.166/0.331
Deviance	1544.434	1861.133	5398.459	3946.211	6083.565
AIC	1586.130	1905.410	5438.311	3960.211	6101.565

Abbreviations: MT, movement time; MU‐APV, movement units after peak velocity; PV, peak velocity; PV1‐b, peak velocity of the first movement unit; TTPV, time to peak velocity.

Models for MU, PV1 and %Dec are reported in Supplemental Table 2.

### 3.3 Effect of Dist, age, and ASD

We found strong evidence of an effect of Dist and age on all kinematic outcomes (*p* < 0.001). Increase in Dist led to longer MT (OR: 1.10, 95% CI 1.09–1.12), larger PV (OR: 1.17, 95% CI 1.15–1.18) and longer TTPV (OR: 1.13, 95% CI 1.11–1.15). Increase in Dist also led to lower odds of PV1‐b (OR: 0.75, 95% CI 0.70–0.81), and greater incidence rate of MU‐APV (IRR: 1.33, 95% CI 1.26–1.41). (Tables 4 and 5). In other words, compared to shorter swipes, we found fewer swipes with peak velocity in the first MU amongst longer swipes. We also found, on average, more MUs after PV in longer swipes.

Increase in 1 year of age led to swipes with shorter MT (OR: 0.71, 95% CI 0.64–0.79), larger PV (OR: 1.25, 95% CI 1.16–1.35), shorter TTPV (OR: 0.74, 95% CI 0.65–0.83), greater odds of PV1‐b (OR: 1.89, 95% CI 1.52–2.35), and reduced the incidence rate of MU‐APV (IRR: 0.61, 95% CI 0.51–0.72).

We found evidence of an effect of ASD for MT, PV, TTPV and MU‐APV, along with interaction effects of ASD × Dist and ASD × age. At the median Dist of 30–40 mm and mean age of 4.7 years, participants in the ASD group had longer MT (OR: 1.13, 95% CI: 0.96–1.33), TTPV (OR: 1.20, 95% CI 1.00–1.44), lower PV (OR: 0.92, 95% CI 0.82–1.03) and greater incidence rate of MU‐APV (IRR: 1.36, 95% CI (1.01–1.83) compared to TD participants. Compared to the TD group, the ASD group had half the odds of PV1‐b (OR: 0.56, 95% CI 0.38–0.82).

### 3.4 Interaction effects

*ASD × Dist*. The ASD *×* Dist interaction for MU‐APV (IRR: 0.91, 95% CI 0.84–0.99), indicating a smaller effect of ASD at longer Dist and a smaller effect of Dist for the ASD group compared to the TD group.

*ASD × Age*. ASD *×* age interactions for MT, PV and MU‐APV show that the effect of ASD became larger with an increase in age and the effect of age was smaller for the ASD compared to TD group. Compared to the TD group, the age‐attributed reduction in kinematic outcome for the ASD group was smaller for MT (OR: 1.33, 95% CI 1.11–1.60), TTPV (OR: 1.21, 95% CI 0.99–1.48) and MU‐APV (IRR: 1.35, 95% CI 1.02–1.80); and age‐attributed increase in PV was smaller (OR: 0.88, 95% CI 0.77–1.00).

### 3.5 Correlation analysis

PV1 was strongly positively correlated with PV (*r* = 0.87) and showed the same patterns of correlations: negatively correlated with TTPV, MT and MU, and weak to no correlation with %Dec. MU was more strongly correlated with PV1 (*r* = −0.40) than PV (*r* = −0.19). PV1‐b was positively correlated with PV1 (r = 0.51) but only weakly correlated with PV (*r* = 0.19), and otherwise showed the same pattern of correlations with PV1. MU‐APV was positively correlated with MU (*r* = 0.75), %Dec (*r* = 0.46) and MT (*r* = 0.51), and showed no correlation with TTPC, PV1 and PV. PV1‐b was not correlated with MU‐APV (Figure 7).

MU and MT were strongly positive correlated (*r* = 0.72) and showed the same pattern of correlations with other kinematic variables: positively correlated with TTPV and MU‐APV, no correlation with %Dec, and negative correlations with PV and PV1 (Figure 7).

### 3.6 Sensitivity analysis

A total of 3684 swipes (93.8% of the analysis sample), 2435 (93.9%) and 1249 (93.7%) from ASD and TD group participants, respectively, met the stricter criteria for sensitivity analysis. Analysis of this dataset produced comparable coefficient estimates (Supplemental Table 3). The ASD × age effect reduced slightly for MU‐APV (IRR: 1.26, 95% CI 0.94–1.69) and increased slightly for PV (OR: 0.82, 95% CI 0.72–0.93).

## 4 DISCUSSION

In line with our hypotheses, we found evidence that (1) children with an ASD diagnosis differed from neurotypical controls in movement kinematics of goal‐directed movements, (2) that, with the exception of PV1‐b, these group differences become larger amongst older children, and (3) that ASD diagnosis influenced the relationship between target distance and movement kinematics related to feedback control.

### 4.1 Swipe kinematics and motor control processes in smart‐tablet gameplay

First, we discuss the rationale for deriving the two additional kinematic variables (PV1‐b and MU‐APV) in the context of our study paradigm and their suitability.

*Movement kinematics reflect planning and adjustment in the final step of a two‐step (1) reach‐to‐food and (2) food‐to‐plate movement*. The kinematic structure of swipe movements contains a primary transport unit in which PV occurs, the magnitude of which relates to feedforward movement planning to cover the target distance; but whether PV occurred in the first MU (and hence resulting in a greater PV1) depended on successful action chaining—that is, whether the movement plan was available following the initial reach‐to‐contact to next immediately execute the second food‐to‐plate movement. Feedback processes act later to produce corrective movements altering the movement plan, appearing in the kinematic profile as MUs after the PV.

*%Dec*. %Dec is typically used to analyse smooth movements consisting of a single movement peak. %Dec is sensitive to variation in different aspects of the kinematic profile, that is, the variation in MT before or after PV and the MU in which PV occurs. The effect of ASD or age on %Dec might have been cancelled out as each predictor can have unique effects on different aspects of the movement profile, which can in turn alter the value of %Dec in opposing directions.

*PV1‐b*. This binary variable, measuring whether PV was found in the first MU, was based on the above reasoning on the kinematic structure of the two‐step movement. Correlation patterns indicate that it is a good candidate measure of successful action chaining: PV1‐b was only weakly correlated with PV suggesting that it is distinct from processes involved in generating the feedforward movement plan.

*MU‐APV*. This variable was a count of the number of MUs after PV, derived to capture feedback control processes where the movement is set within an action chain of more than one movement step. Positive correlations with %Dec and MT suggest that it is related to decelerative processes towards the movement goal that increase overall MT. Moreover, it appears specific to feedback processes, as it was not correlated with variables associated with feedforward planning and control processes (PV, PV1, PV1‐b, TTPV).

*Relationship to other kinematic parameters*. Our study focused on ‘local’ landmarks in the movement profile of specific goal‐directed actions, grounded in a theory of prospective motor control, to make inferences on specific sub‐second processes implicated in voluntary movement generation. Other kinematic variables shown to be different in autism include ones also related to these landmarks, such as their spatiotemporal variability (Foster et al., 2020b; Glazebrook et al., 2006). Others focus on more ‘global’ features, such as the jerk amplitude averaged over repetitions of movement (Cook et al., 2013), amount of speed fluctuations (s‐peaks) within cycles of movement (Wu et al., 2018) or the straightness in movement path (Weisblatt et al., 2019), all of which likely arise from the coordination of multiple processes over space and time.

A standard battery of kinematic parameters for measuring motor differences in autism is conceivable and has functional utility in the development of algorithmic assessment of movement (Millar et al., 2019; Wedvan et al., 2019; Lidstone et al., 2021). Such a battery, incorporating parameters with neurobiological and developmental significance, could be tailored to suit specific aims with parameter selection guided by data type and context. For example, more ‘global’ kinematic features may be more useful for continuous, long time‐scale, accelerometer data acquired in naturalistic environments, whereas analysis of local features might be more suitable when acquired under experimental or structured conditions. Ultimately, a combination of kinematic features of the two types may computationally characterise an ‘autism motor signature’ useful for precise digital phenotyping or computational solutions to support early screening, clinical diagnosis and therapeutic monitoring, and for research purposes (Hocking & Caeyenberghs, 2017).

### 4.2 Movement kinematic differences between ASD and TD

In the remaining discussion, we refer to results related to the kinematic outcomes MT, PV, TTPV, PV1‐b and MU‐APV.

In line with the general trend reported in previous work (Campione et al., 2016; Forti et al., 2011; Glazebrook et al., 2006; Mari et al., 2003; Yang et al., 2014), we found longer MT and TTPV in autistic children compared to neurotypical controls, and also lower PV and fewer MUs in swipes made by autistic children. Our findings further indicate that the extent of kinematic differences between ASD and TD was larger amongst older children.

There are a number of reasons why previous studies reported findings different to ours. First, our findings may be specific to autistic children with more severe difficulties, as our sample comprised children presenting at specialist clinics. In contrast, some studies, which did not find group differences between autistic individuals and controls in PV or MT, included only children with high functioning autism or Asperger's Disorder (Papadopoulos et al., 2012), or specifically excluded individuals with low cognitive functioning (Campione et al., 2016; Yang et al., 2014). This explanation is supported by evidence that group differences in PV were modulated by level of functioning (Mari et al., 2003), and PV was positively correlated with IQ in both groups (Forti et al., 2011). Second, insufficient power could explain why group differences were not found in other studies. Movements, particularly in childhood are characterised by variability (Thelen & Smith, 1994), and our data support this (Figure 3). There is high within‐individual variability as children do not always perform movements with the same kinematic characteristics. Including a large range of ages in relatively small sample sizes (Dowd et al., 2012), or using a two‐step task can introduce further between‐individual variability that makes it difficult to detect group differences. In contrast, variability is likely less problematic in studies of young adults, which have found group differences (Glazebrook et al., 2006). Third, developmental changes amplifies group differences, as indicated by the ASD × age interaction in our study. This can explain why there was only a trend towards lower PV in autistic compared to neurotypical children in Forti and colleagues (2011) study of young children age 3–4 years old. Finally, task differences test different movement strategies and may alter group differences. Although Cook and colleagues (2013) found greater PV and shorter MT in autistic young adults, this is likely restricted to the context of repetitive unconstrained arm movements they investigated, in contrast to our study and the majority of related work, which focused on goal‐directed movements.

**FIGURE 4:**
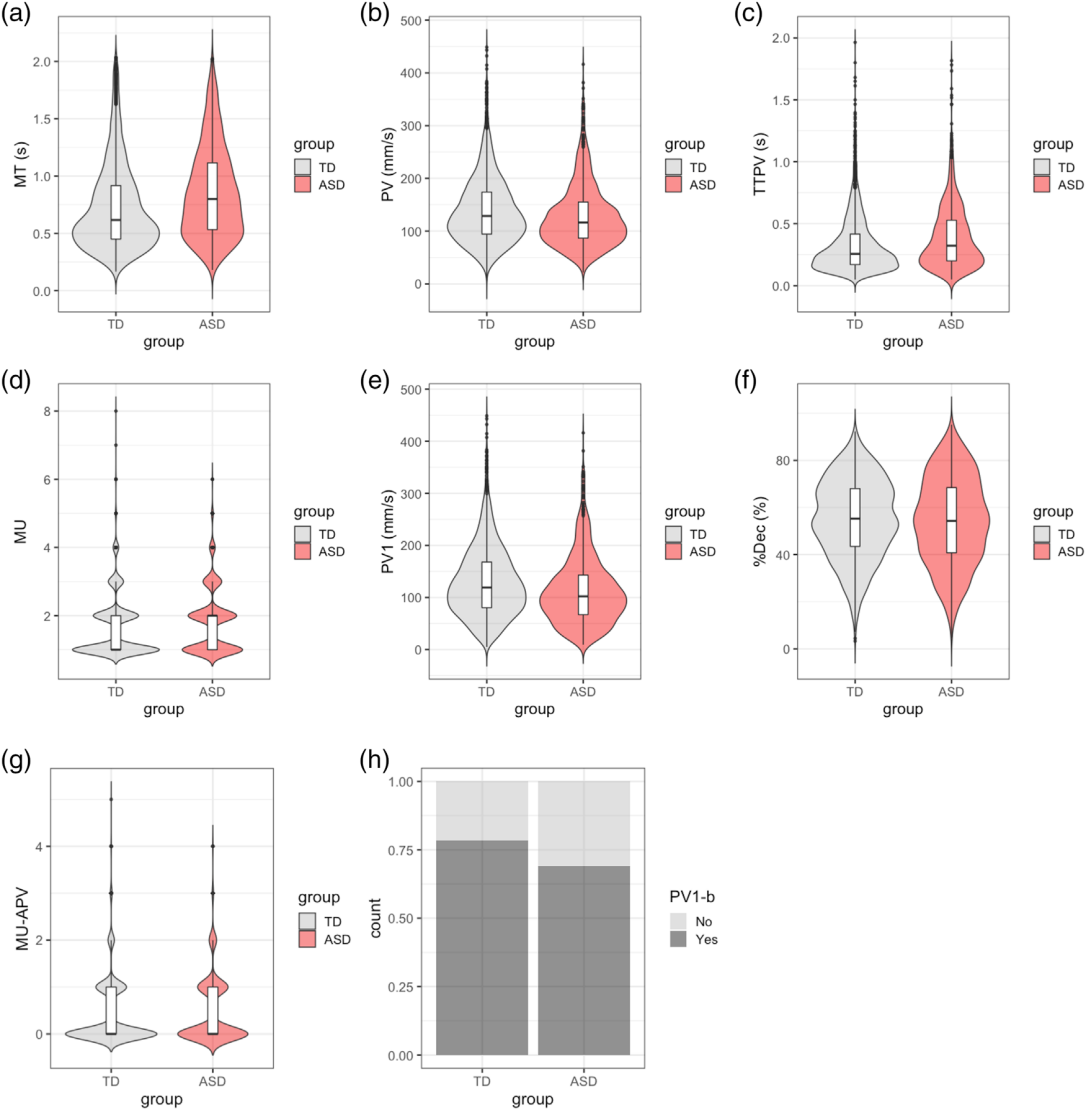
(Top) Descriptive plots of kinematic variables (a priori) across the analytic sample of 3926 swipes. From a‐f: Violin plots of MT, PV, TTPV, MU, PV1, %Dec, by ASD diagnosis (Black: TD, Red: ASD); (Bottom) Descriptive plots of kinematic variables (exploratory) across the analytic sample of 3926 swipes. g: Violin plot of MU‐APV by ASD diagnosis (Black: TD, Red: ASD); h: Barplot of PV1‐b showing proportions (relative counts) of Peak Velocity occurring in the first movement unit (Yes) or occurring in subsequent movement units (No), for swipes made by each group [Corrections made on 21 December 2021, after first online publication: Figure part labels for 4g and 4h have been corrected in this version.]

**FIGURE 5:**
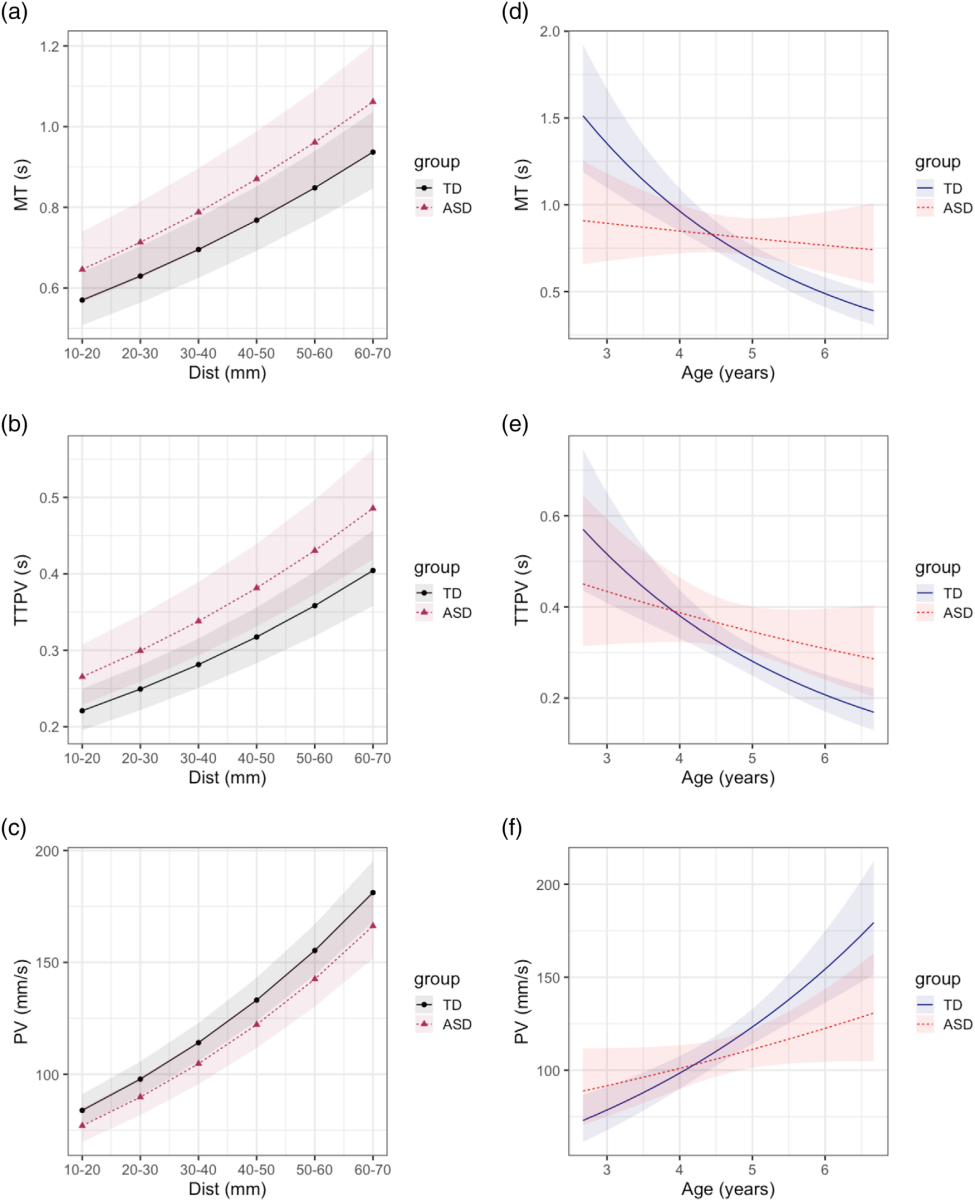
Predicted marginal effects of mixed effect models. *Top to bottom*: movement time (MT), time to peak velocity (TTPV), peak velocity (PV). (a)–(c) Effect of target distance by ASD diagnosis at grand mean of age (4.7 years). (d)–(f) Effect of age by ASD diagnosis at grand median category of target distance (30–40 mm**)**. No effect of ASD × Dist was found ((a)–(c) but ASD × age cross‐over interaction effects show diverging trends in these movement kinematics and differing effects of ASD diagnosis (d)–(f)

**FIGURE 6:**
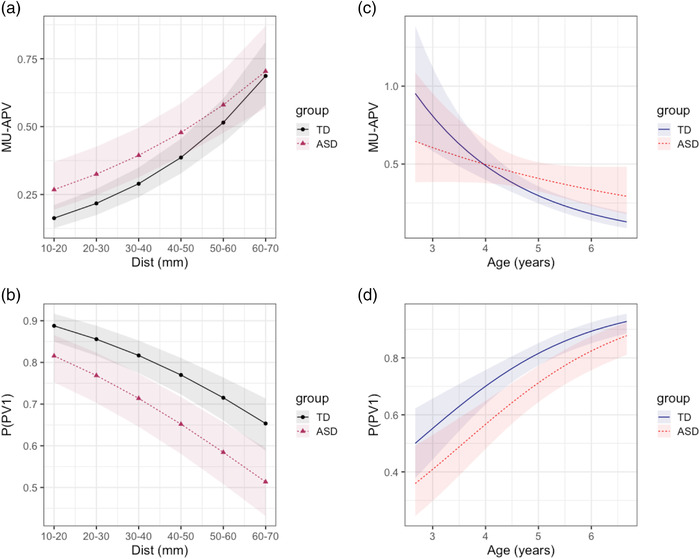
Predicted marginal effects of mixed effect models. *Top*: movement units after peak velocity (MU‐APV) (expected value); *bottom*: peak velocity of the first movement unit (PV1‐b) with predicted probabilities. *Left*: Effect of target distance by ASD diagnosis at grand mean of age (4.7 years). *Right*: Effect of age by ASD diagnosis at grand median category of target distance (30–40 mm). The ASD × Dist for movement units after peak velocity (MU‐APV) show that for further targets, the ASD group did not show an increase in number of MU‐APV to the same extent as the TD group, while ASD × age effect shows that the ASD group execute movements with greater MU‐APV, but this effect is only seen amongst older children (a, c). No interaction effects were found for PV1‐b, indicating that the effect of ASD does not change with age or Dist (b, d)

**FIGURE 7:**
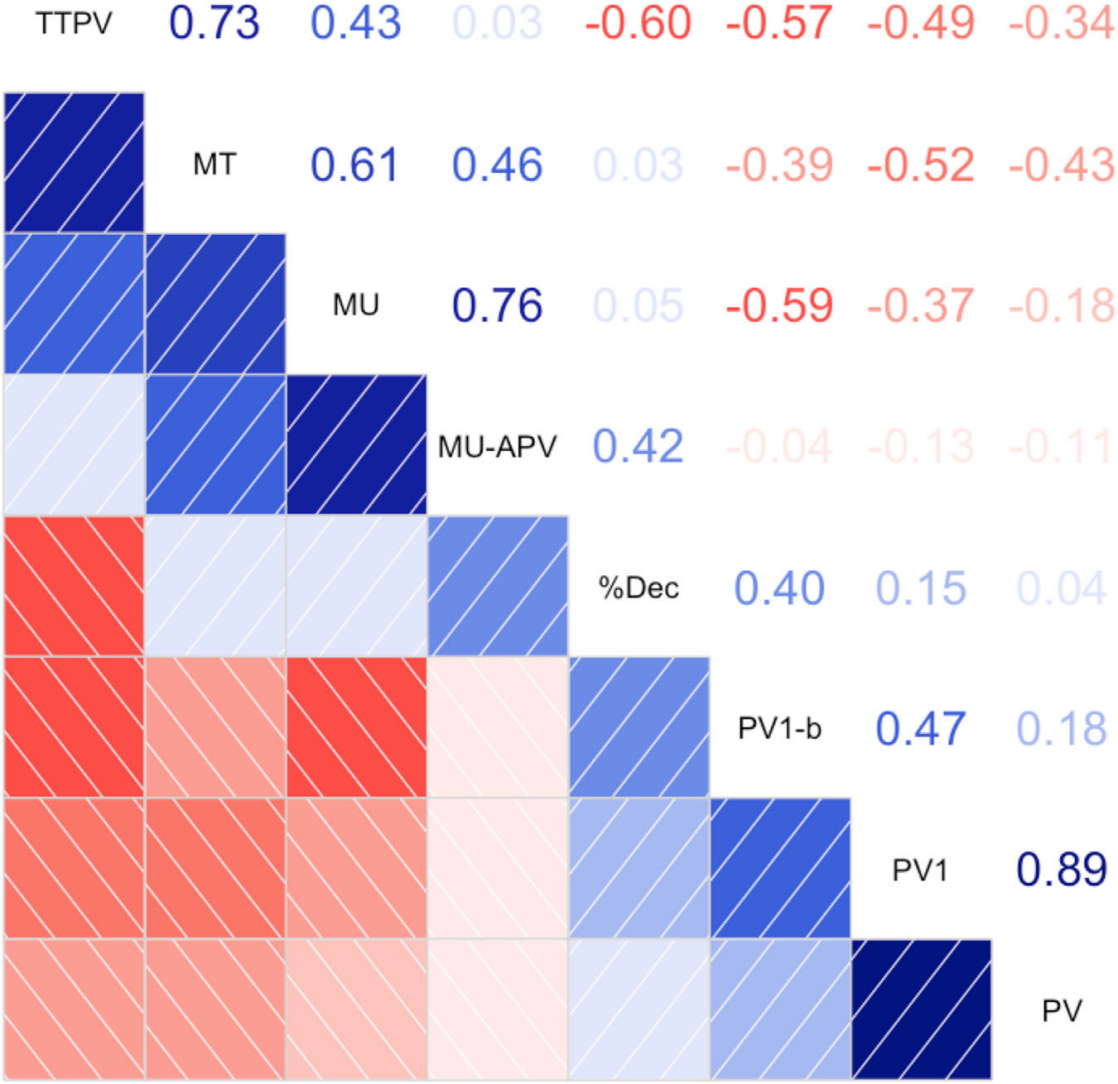
Correlation matrix for a priori kinematic outcomes time to peak velocity (TTPV), movement time (MT), movement unit (MU), deceleration phase (%Dec), movement units after peak velocity (MU‐APV), peak velocity of the first movement unit (PV1‐b), peak velocity of the first movement unit (PV1), peak velocity (PV). Pairwise Pearson correlations are shown in the right‐diagonal panel and direction/strength of correlation in the left‐diagonal panel. Blue indicates positive correlations and red indicates negative correlations, and greater saturation indicates stronger correlations

*Modulating control processes with task difficulty*. Increase in target distance increases hence task difficulty. In line with motor control theory, this led to an increase in MT and PV, as well as a decrease in TTPV and PV1‐b in both groups. Target distance is thought to have little influence on feedback control processes in studies of smooth movements in adults, which typically consist of a single movement peak (Bootsma et al., 2004; MacKenzie et al., 1987). We present new evidence that the number of MU‐APV in children's goal‐directed swipes increases with target distance. One explanation may be that greater target distance increases the opportunity for error and therefore greater use of corrective movements. However, every 1‐year increase in age also halves the number of MU‐APV in children's goal‐directed swipes. This suggests that development of successful feedforward control with age reduces the need to recruit subsequent feedback processes. Further, the ASD × Dist interaction suggests that at smaller target distances, the TD group is able to use predominantly feedforward control and rely less on corrective feedback movements, unlike the ASD group. These group differences disappear at longer target distances as the TD group recruits similar extents of feedback control as the ASD group to overcome the greater task difficulty. Our findings overall, suggest that feedforward control processes are intact in autistic children but less effective than neurotypical controls, thereby resulting in a greater reliance on feedback control.

*Developmental differences in movement kinematics*. Reduction in MT, TTPV and MU‐APV, and increase in PV in TD children is consistent with what we expect with motor development. With development, feedforward processes become more efficient and accurate, as represented by increasing dominance of a single, primary transport unit and fewer subsequent feedback phases to correct the trajectory to the target (Berthier & Keen, 2006; von Hofsten, 1991). Our finding that PV became more likely to occur in the first MU with age likely indicates that action chaining becomes more consistent with motor development. Interaction effects show a smaller reduction in MU‐APV in the ASD group with increase in age compared to the TD group alongside lower PV, longer TTPV and longer MT, and this evidence supports a previous explanation for why ASD and TD groups have different movement kinematics, that autistic children develop different movement strategies compared to neurotypical controls (Glazebrook et al., 2006; Mari et al., 2003). Research using a computational perspective suggests that reducing the magnitude of PV is an optimal strategy to compensate for noise during motor execution, thereby reducing the error resulting from feedforward control processes (Harris & Wolpert, 1998). An earlier study focusing on variability in feedforward processes also supports the idea that autistic individuals reduce PV to minimise the effects of noise on the ongoing movement (Glazebrook et al., 2006). In line with findings relating to the ‘low ability’ ASD group in Mari and colleagues’ (2003) study, our findings show that the autistic children in our sample, recruited from specialist clinics, rely more on feedback processes to reach the movement goal, that is, using corrective submovements.

*Sensorimotor integration*. Group differences in the developmental trends of feedforward and feedback movement kinematics may be attributed to differences in the development of sensorimotor integration. Integration of vision and proprioception is important for planning movement as well as to direct ongoing movement towards its goal as it unfolds. Proprioceptive functioning improves substantially around age 4–5 years (Chicoine et al., 1992; von Hofsten & Rösblad, 1988) and continues during childhood (King et al., 2010). This can, in turn, contribute to improved integration of visuo‐proprioceptive information relating the body to the movement goal to enable more accurate predictive and online control processes (Babinsky et al., 2012). Smaller increases in PV with age and smaller reductions in MUs after PV in the ASD group relative to controls is in line with what we would expect if the development of sensorimotor integration was disrupted in autism. Indeed, research appears to be converging on a disruption in sensorimotor integration in autism (Gowen & Hamilton, 2013; Hannant et al., 2016), affecting how multisensory information is used for motor planning (Paton et al., 2012), execution and online control (Glazebrook et al., 2009; Schmitz et al., 2003), and motor learning (Haswell et al., 2009; Izawa et al., 2012; Marko et al., 2015; Sharer et al., 2016).

*Action chaining*. Our findings on PV1‐b support earlier reports that action chaining is affected in autism. Movements in the ASD group were roughly half as likely to contain the primary transport unit as the first MU than in the TD group, suggesting that each second action step is more likely to be performed independently of the first (Cattaneo et al., 2007; Fabbri‐Destro et al., 2009). This may result from a difficulty in incorporating the intention of the final motor act within an action chain (Cattaneo et al., 2007; Fabbri‐Destro et al., 2009). However, we also found that increase in target distance reduced the action chaining performance of both groups to the same extent, in line with evidence that ASD children were able to modulate grasp height of the first movement step based on the target height (Ansuini et al., 2018). At first glance, the latter finding appears to contradict existing explanations that differences in the kinematics of chained actions are due to differences in incorporating the intention of the final motor act. In fact, this finding may indicate more subtle differences in planning processes to achieve movement goals in autism—specifically, that (low‐level) visuospatial characteristics of the goal are successfully incorporated in action chaining even if other contextual aspects of the final goal are not.

Given limited research on action chaining in ASD, our explanation highlights an important area for future research. Ansuini and colleagues’ (2018) study was the only one, which included a task to investigate whether modulation of the first movement step was influenced by the social context, that is, the partners’ intention. However, their task was not sensitive enough to detect modulation of movement in either the TD or ASD group. Further research could clarify how action chaining is disrupted in autism, whether the difficulty lies in incorporating contextual and intentional information about the final goal rather than low‐level visuospatial aspects. This can also inform whether differences in movement kinematic organisation might have implications beyond the motor domain to affect intentional anticipation and intentional understanding in social contexts, potentially contributing to socio‐cognitive difficulties characteristic of autism (Trevarthen & Delafield‐Butt, 2013; Cook, 2016).

*Deficit versus strategic optimisation*. Our study adds to the debate on whether kinematic differences indicate simply a disturbance in the sensorimotor system in autism, or a strategic optimisation due to underlying differences in the neuromotor system (Elliott et al., 2020; Latash & Anton, 1996). In previous studies, it was difficult to argue for whether atypical kinematic differences relate to the former or latter in older individuals whose motor skills are already well‐developed. In contrast, our study shows that kinematic differences between groups can be observed as early as preschool years. Crucially, these group differences begin to be apparent around a time when visuo‐proprioceptive integration is developing. This allowed us to reason that sensorimotor development under different constraints (sensorimotor integration) might be what perpetuates kinematic differences, reflecting different adaptive neuro‐ and psycho‐ motor strategies in autism.

### 4.3 Strengths, limitations and future directions

To our knowledge, this is the largest study of movement kinematics in autism—studies of motor control kinematics typically consist of around 20 individuals per group (e.g. in studies by Glazebrook and colleagues (2006, 2009)). This step‐increase in population size was enabled by smart device and ecological gameplay assessment. While ecological gameplay allows for behavioural variability, this was balanced by acquisition of a large number of repeated measurements across participants to enable a high‐powered analysis in a mixed‐effects regression model. We have embraced the benefits of smart‐tablet technology for its ease of large‐scale data collection and use outside an experimental environment; however, this is not without compromise, and our findings should be considered in light of its limitations.

Importantly, the ‘Sharing’ game did not have an explicit speed or accuracy requirement: children moved food pieces to within the perceptual boundaries of the plate and the task was considered successful as long as movement ended within the end area. Although these food‐to‐plate movements still showed a speed‐accuracy trade‐off indicating that children followed the task demands to make movements efficiently and accurately, we may have underestimated group differences. This is because children in the ASD group may still be able to complete the task without being as accurate—in earlier studies, differences relative to controls in MUs were greater when the task required greater accuracy (e.g. to smaller targets) (Forti et al., 2011), and in an unconstrained movement task, autistic adults showed greater movement velocity, but tended to overshoot more (Cook et al., 2013).

Using a commercially developed game also meant that we could not incorporate experimental parameters of interest. First, we derived target distance from the recorded properties of the resulting movement, that is, the straight‐line distance between the points at which a touch began and ended. However, to account for the possibility that the resultant movement can deviate from the initial movement plan, we increased the validity of our definition of target distance using categories of target distance. Second, we were unable to assess endpoint accuracy in our study as children were able to complete the task by finishing the movement within the plate and cartoon character area. This meant that, even though they were likely to be aiming at the clearly demarcated plate boundaries, control errors during the movement were tolerated by the gameplay design. As such we could not evaluate whether the different kinematic strategies used by each group had the same level of success.

Our study only used a cross‐sectional design to study age trends, but has highlighted the importance of investigating the longitudinal development of movement kinematics in motor development. While this study was in‐part aimed at assessing the suitability of kinematic analysis on movements sampled during smart‐tablet gameplay and effects should be considered in light of this exploratory aspect, our study shows the feasibility of sampling and analysing goal‐directed movements made in a gameplay context. This approach is particularly suitable for studying movements in young children with autism as it does not require extensive instructions and can be used outside strict laboratory environments, such as in schools and clinics (Anzulewicz et al., 2016; Millar et al., 2019).

Finally, future research should focus on mapping the relationship between motor kinematic, feedforward and feedback differences with learning, cognition and psychological development in autism. Direct evidence on these associations will strengthen the arguments we have provided for a disruption to prospective movement in autism and its importance for learning and development.

## 5 CONCLUSIONS

Our study demonstrates the use of kinematic analysis on movements sampled on a smart device touchscreen, during ecological serious gameplay. We show differences in the movement kinematics of autistic children compared to neurotypical controls, of longer MT and TTPV, lower PV, fewer occurrences of PV in the first MU and greater number of MUs. We further report age‐dependent differences in movement kinematic organisation between the two groups, as a result of different developmental trends. From these findings, we conclude that autism affects the involvement of both predictive feedforward processes and corrective feedback processes to achieve efficient goal‐directed movement. Our findings suggest that feedforward control processes are intact, but less effective in autistic than neurotypical children, resulting in a greater reliance on feedback control. This points to fundamental differences in the underlying neuromotor organisation and integration of perceptuomotor information to anticipate, prepare and enact a self‐generated movement to achieve a desired goal, with implications for children's cognitive development, and learning.

## CONFLICT OF INTEREST

The authors declare no conflict of interest in this work.

## AUTHOR CONTRIBUTIONS

Yu W. Chua conceptualised and designed the investigation and analysis of study data, processed, analysed and interpreted the data, drafted and revised the manuscript. Szu‐Ching Lu contributed to conceptualisation, data processing and data description. Anna Anzulewicz, Krzystof Sobota and Jonathan Delafield‐Butt designed the study, including ethical permissions. Anna Anzulewicz contributed data acquisition. Philip Rowe, Christos Tachtatzis, Ivan Andonovic and Jonathan Delafield‐Butt supervised data analysis and interpretation of data. All authors contributed authorship to revisions of the manuscript. All authors approved the final version of the manuscript and are accountable for the accuracy and integrity of the manuscript.

## Supporting information



Supporting Information

## Data Availability

Derived data and analysed data supporting the findings of this study are openly available in Open Science Framework at https://osf.io/xjdf8/. Cite as: Chua, Y. W., Lu, S. C., Anzulewicz, A., Sobota, K., Tachtatzis, C., Andonovic, I., Rowe, P., Delafield‐Butt, J. (2021, May 27). Ipad Swipe Kinematics. Retrieved from osf.io/xjdf8
