# Supplementary material for: Developmental differences in the prospective organisation of goal‐directed movement between children with autism and typically developing children: A smart tablet serious game study
Source: Dev Sci. 2021 Dec 6;25(3):e13195. doi: 10.1111/desc.13195 (PMC9287065; doi:10.1111/desc.13195)
Supplement: Supplementary file 1 — Supporting Information [file DESC-25-0-s001.docx]

**Supplementary material**

**Developmental Differences in the Prospective Organisation of Goal-Directed Movement Between Children with Autism and Typically Developing Children: A Smart Tablet Serious Game Study**

**Supplemental Methods**

**Selection of filter frequency**

4-8 Hz frequency filters are commonly used in human movement analysis (Bartlett, 2007) and an 8 Hz filter was chosen based on comparison of 100 randomly chosen position vectors filtered at 4, 6 and 8 Hz. Filtering at 8hz had minimal or no perceptible distortion of signals, and reducing the frequency further to 6Hz and 4Hz led to perceptible and increasing distortion of coordinate profiles. After filtering x and y position vectors, x and y velocity vectors were obtained through numerical differentiation using the five-point stencil (Abramowitz & Stegun, 1964). This method allows more accurate derivatives as noise in data can be amplified as a result of finite differentiation, and has been applied to finger movement position data (Rachaveti et al., 2018).

**Model building**

A top-down model building approach was used, first fitting the full model with all random intercepts, random slopes and fixed effects (including interaction effects) (Model Re1). In Step 2, using the full model, we determined if a random slope should be included, both fitted using the REML estimator (Model Re2). A random slope was included if Akaike Information Criterion (AIC) was lower in Model Re1. In Step 3, using the random effects structure optimised in Step 2, the fixed effects structure was optimised by fitting models using the Full Information Maximum Likelihood estimator with an interaction effect removed at each step and nested models compared using a likelihood ratio test (Models Fe1-4). Inclusion of interaction effects was guided by effect estimates and likelihood ratio tests. Models fitted in the model building procedure (Re1-2, Fe1-4) are reported in the Supplemental Tables 4 and 5.

Models were tested for residual normality, homogeneity of variance and linearity, by examining residual q-q plots, scatter of residuals against fitted values, and scatter of residuals against observed values, respectively. MU was visually inspected to follow a Poisson distribution and the final model was checked for overdispersion.

**Supplemental Table 1.
Full breakdown of swipes excluded according to exclusion criteria.**

|  | **Total n(%total)** | **TD  n(%total)** | **ASD n(%total)** |
| --- | --- | --- | --- |
| **Food-to-Plate swipes** | 4917 (100%) | 3233  (65.8%) | 1684  (34.2%) |
| **Not suitable for analysis** | 159  (3.2%) | 50  (31.4%) | 109  (68.6%) |
| **Non-task-conforming swipes** | 832  (16.9%) | 590 (70.9%) | 242 (29.1%) |
| Swipes from excluded participants (criteria: <10% total swipes comprising food-to-plate swipes^†^) | 55  (1.1%) | 22  (40%) | 33  (60%) |
| No movement units | 265 (5.3%) | 221 (83.4%) | 44 (16.6%) |
| Movement Time outliers (criteria: >2.0s) | 186 (3.8%) | 149 (80.1%) | 37 (19.9%) |
| Target Distance outliers (criteria: 10mm<Dist<70mm^‡^) | 80 (1.6%) | 56 (70.0%) | 24 (30.0%) |
| Straightness ratio outliers (criteria: >1.5) | 246 (5.0%) | 142 (57.7%) | 104 (42.3%) |
|  | **Total n(%total)** | **TD  n(%total)** | **ASD n(%total)** |
| **Total swipes excluded** | 991  (20.2%) | 640 (64.6%) | 351 (35.4%) |
| **Analysis sample (Goal-directed swipes)** | 3926 (79.8%) | 2593  (66.0%) | 1333  (34.0%) |

^†^swipes from 11 participants were excluded

^‡^only 1 swipe with Dist<10mm

**Supplemental Figure 1A-1B. Model diagnostics (Linear mixed-effect models).**

**A: (Right to left) Normality of residuals (Quantile-quantile plot and density plot) and homoscedasticity of residuals**

Top to bottom: Model diagnostics for log-transformed MT, PV and TTPV


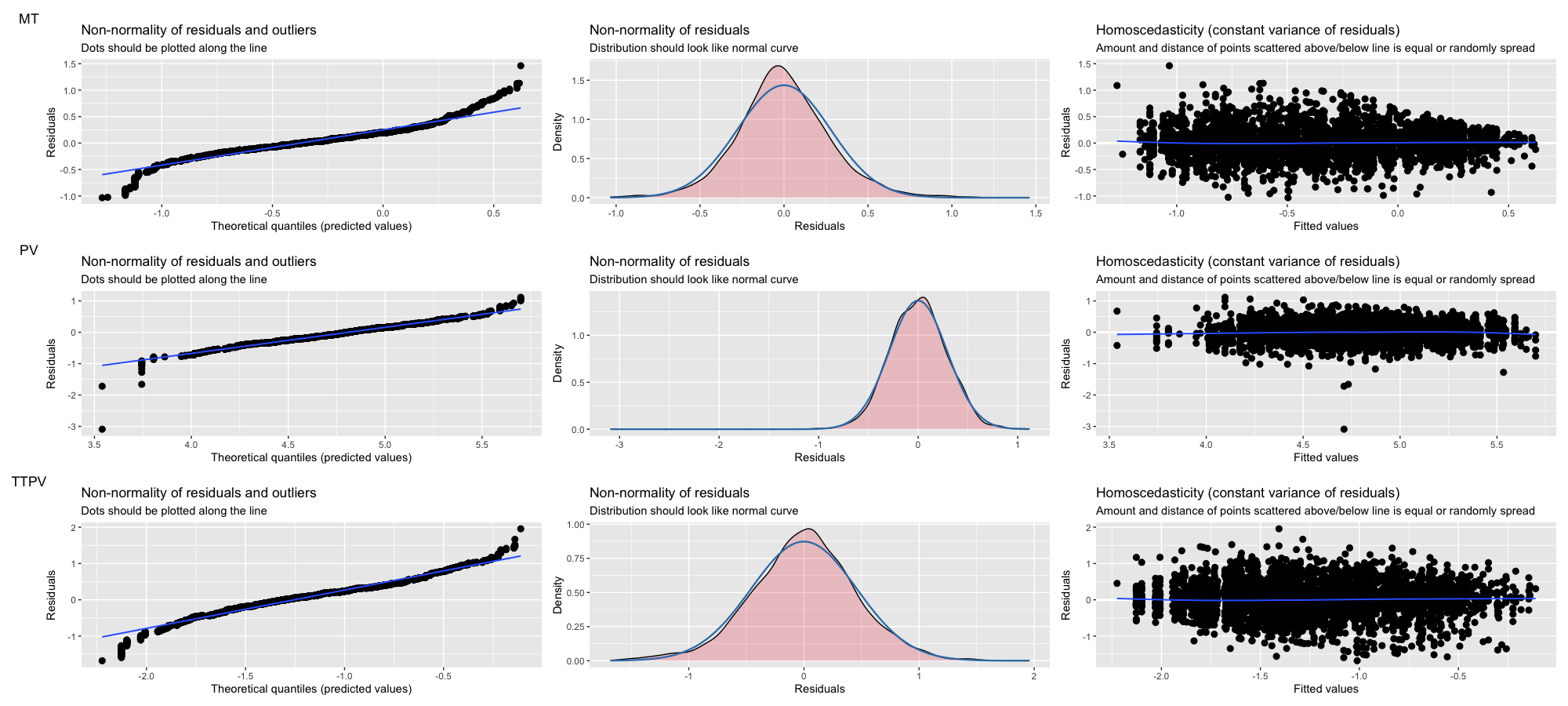


**B: Linearity**Top to bottom: Model diagnostics for MT, PV, TTPV

**
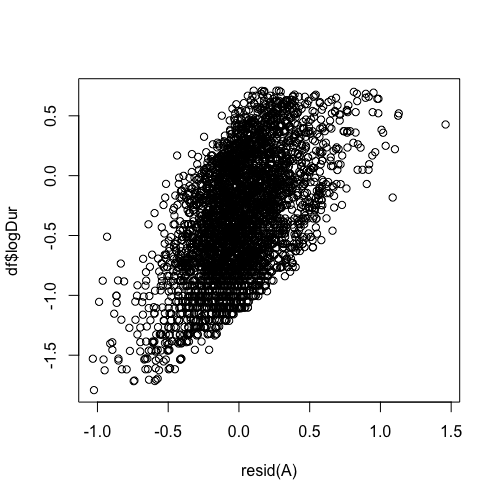
**

**
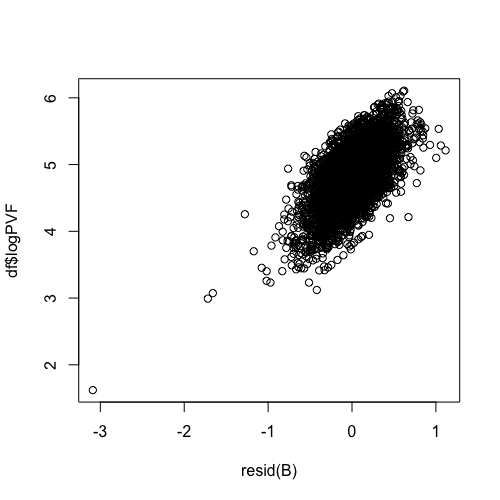
**

**
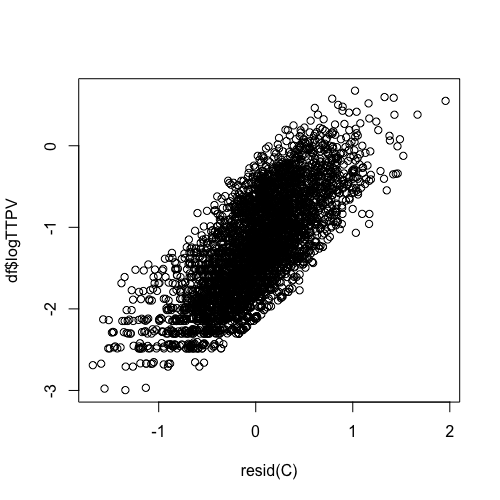
**

**Supplemental Table 2. Final Models.**

**Zero-truncated Poisson and Linear Mixed Effect Movement Units, Peak Velocity 1MU, Deceleration Phase.** The model for Peak Velocity 1MU did not meet assumption for homogeneity of variance and was the only model where including Target distance x Age improved model fit. 16.6% of the total variance was attributed to participant variation, and conditional on this, fixed effects explained only 1% of the total variance.

|  | **Movement Units** | **PV1 (mm/s)** | **%Dec (%)** |
| --- | --- | --- | --- |
| ***Fixed effects*** | *Incidence Rate Ratios (95% CI)* | *Coefficient  (95% CI)* | *Coefficient  (95% CI)* |
| Intercept | 0.86  (0.73 – 1.01) | 116.34 ^***^ (106.78 – 125.90) | 55.62 ^***^ (53.52 – 57.72) |
| Target Distance | 1.26 ^***^ (1.21 – 1.31) | 14.74 ^***^ (12.86 – 16.63) | -0.78 ^**^ (-1.32 – -0.24) |
| Age | 0.63 ^***^ (0.54 – 0.72) | 24.54 ^***^ (15.94 – 33.14) | 1.38  (-0.39 – 3.15) |
| ASD | 1.43 ^**^ (1.11 – 1.85) | -15.35 ^*^ (-29.36 – -1.34) | -2.09  (-5.33 – 1.15) |
| ASD x Age | 1.32 ^*^ (1.04 – 1.68) | n.a | n.a |
| ASD x Target Distance | 0.94 ^*^ (0.88 – 0.99) | n.a | n.a |
| Target Distance x Age | n.a | 4.89 ^**^ (2.78 – 6.99) | n.a |
| ***Random Effects*** | |  | |
| σ^2^ | 0.18 | 2169.57 | 254.89 |
| τ_00_ _Subject_ | 0.24 | 1044.46 | 44.57 |
| τ_11_ _Subject. Target distance_ | 0.00 | 39.44 | 2.56 |
| ρ_01_ _Subject_ | -0.91 | 0.66 | -0.41 |
| Observations | 3926 | 3926 | 3926 |
| Marginal R^2^ / Conditional R^2^ | 0.364 / 0.721 | 0.236 / 0.509 | 0.012 / 0.166 |
| Deviance | n.a | 41574.682 | 33090.719 |
| AIC | 7991.987 | 41574.333 | 33101.574 |
| ** p<0.05   ** p<0.01   *** p<0.001* | | | |

**Supplemental Table 3. Sensitivity analyses.**

**Mixed effects models for Movement Time, Peak Velocity, Time to Peak Velocity, Peak Velocity 1MU-b, Movement Units APV.** In sensitivity analysis we reran the final models on a stricter sample including only swipes that began with an acceleration phase or with the first velocity minima occurring within 5mm of contacting the touchscreen surface, and ended with a deceleration phase or reaching a velocity minima within 5mm of touch ended. This produced comparable parameter estimates across the models. Notably, this slightly altered the effect estimates for the ASD x Age effect on PV and MU-APV.

|  | **MT (s)** | | **PV (mm/s)** | | **TTPV (s)** | | **PV1-b** | | **MU-APV** |
| --- | --- | --- | --- | --- | --- | --- | --- | --- | --- |
| ***Fixed effects*** | *Coefficient (95% CI)* | *Coefficient  (95% CI)* | | *Coefficient (95% CI)* | | *Odds Ratios (95% CI)* | | | *Incidence Rate Ratios  (95% CI)* |
| Intercept | 0.70 ^***^ (0.63 – 0.78) | | 114.40 ^***^ (106.24 – 123.19) | | 0.28 ^***^ (0.25 – 0.32) | | 4.35 ^***^ (3.34 – 5.68) | | 0.30 ^***^ (0.25 – 0.37) |
| Target Distance | 1.10 ^***^ (1.09 – 1.12) | | 1.17 ^***^ (1.15 – 1.18) | | 1.13 ^***^ (1.11 – 1.15) | | 0.75 ^***^ (0.70 – 0.81) | | 1.32 ^***^ (1.25 – 1.39) |
| Age | 0.71 ^***^ (0.64 – 0.79) | | 1.26 ^***^ (1.16 – 1.35) | | 0.74 ^***^ (0.65 – 0.83) | | 1.92 ^***^ (1.54 – 2.41) | | 0.61 ^***^ (0.52 – 0.73) |
| ASD | 1.14  (0.97 – 1.34) | | 0.95  (0.85 – 1.06) | | 1.20  (1.00 – 1.43) | | 0.55 ^**^ (0.37 – 0.82) | | 1.36 ^*^ (1.01 – 1.82) |
| ASD x Age | 1.31 ^**^ (1.09 – 1.57) | | 0.82 ^**^ (0.72 – 0.93) | | 1.22  (1.00 – 1.50) | | n.a | | 1.26  (0.94 – 1.69) |
| ASD x Target distance | n.a | | n.a | | n.a | | n.a | | 0.91 ^*^ (0.84 – 1.00) |
| ** p<0.05   ** p<0.01   *** p<0.001* | | | | | | | | | |
|  | **MT (s)** | | **PV (mm/s)** | | **TTPV (s)** | | **PV1-b** | | **MU-APV** |
| ***Random effects*** | *Estimates* | *Estimates* | | *Estimates* | | *Estimates* | | *Estimates* | |
| σ^2^ | 0.08 | | 0.08 | | 0.21 | | 3.29 | | 1.16 |
| τ_00_ _Subject_ | 0.13 | | 0.06 | | 0.13 | | 0.63 | | 0.29 |
| τ_11_ _Subject.TargetDistance_ | 0.00 | | 0.00 | | 0.00 | | 0.03 | | 0.01 |
| ρ_01_ _Subject_ | -0.47 | | -0.44 | | -0.18 | | -0.45 | | -0.78 |
| Observations | 3684 | | 3684 | | 3684 | | 3684 | | 3684 |
| Marginal R^2^ / Conditional R^2^ | 0.302 / 0.736 | | 0.353 / 0.631 | | 0.200 / 0.515 | | 0.125 / 0.269 | | 0.163 / 0.328 |
| Deviance | 1285.348 | | 1450.230 | | 5049.671 | | 3727.794 | | 5819.282 |
| AIC | 1327.093 | | 1494.604 | | 5089.542 | | 3741.794 | | 5837.282 |

**Supplemental Tables 4A – 4H. Model building (Random effects).**

Models Re1 and Re2 for MT, PV, TTPV, and PV1, %Dec, MU-APV, PV1-b, and MU-APV

**A. MT (log-transformed)**

|  | **Re1** | **Re2** |
| --- | --- | --- |
| *Predictors* | *Estimates* | *Estimates* |
| (Intercept) | 0.68 ^***^ (0.61 – 0.76) | 0.69 ^***^ (0.62 – 0.76) |
| TargetDist.c | 1.11 ^***^ (1.10 – 1.13) | 1.11 ^***^ (1.10 – 1.12) |
| Age.c | 0.72 ^***^ (0.65 – 0.81) | 0.74 ^***^ (0.66 – 0.83) |
| ASD.f [ASD] | 1.20 ^*^ (1.00 – 1.42) | 1.18  (0.99 – 1.40) |
| TargetDist.c * Age.c | 0.99  (0.98 – 1.01) | 0.99 ^**^ (0.98 – 1.00) |
| Age.c * ASD.f [ASD] | 1.33 ^**^ (1.11 – 1.60) | 1.26 ^*^ (1.04 – 1.53) |
| TargetDist.c * ASD.f [ASD] | 0.98  (0.96 – 1.00) | 0.98 ^*^ (0.97 – 1.00) |
| **Random Effects** | | |
| σ^2^ | 0.08 | 0.08 |
| τ_00_ | 0.13 _Subject.f_ | 0.12 _Subject.f_ |
| τ_11_ | 0.00 _Subject.f.TargetDist.c_ |  |
| ρ_01_ | -0.48 _Subject.f_ |  |
| ICC | 0.61 | 0.60 |
| N | 71 _Subject.f_ | 71 _Subject.f_ |
| Observations | 3926 | 3926 |
| Marginal R^2^ / Conditional R^2^ | 0.291 / 0.726 | 0.276 / 0.713 |
| Deviance | 1540.544 | 1577.926 |
| AIC | 1601.680 | 1637.438 |
| ** p<0.05   ** p<0.01   *** p<0.001* | | |

**B. PV (log-transformed)**

|  | **Re1** | **Re2** |
| --- | --- | --- |
| *Predictors* | *Estimates* | *Estimates* |
| (Intercept) | 114.73 ^***^ (106.42 – 123.69) | 114.67 ^***^ (106.38 – 123.60) |
| TargetDist.c | 1.16 ^***^ (1.14 – 1.18) | 1.16 ^***^ (1.15 – 1.17) |
| Age.c | 1.24 ^***^ (1.15 – 1.35) | 1.24 ^***^ (1.15 – 1.34) |
| ASD.f [ASD] | 0.90  (0.80 – 1.02) | 0.90  (0.80 – 1.02) |
| TargetDist.c * Age.c | 1.00  (0.99 – 1.02) | 1.01  (1.00 – 1.01) |
| Age.c * ASD.f [ASD] | 0.88  (0.77 – 1.00) | 0.89  (0.78 – 1.01) |
| TargetDist.c * ASD.f [ASD] | 1.01  (0.99 – 1.04) | 1.01  (1.00 – 1.03) |
| **Random Effects** | | |
| σ^2^ | 0.09 | 0.09 |
| τ_00_ | 0.06 _Subject.f_ | 0.06 _Subject.f_ |
| τ_11_ | 0.00 _Subject.f.TargetDist.c_ |  |
| ρ_01_ | -0.32 _Subject.f_ |  |
| ICC | 0.41 | 0.40 |
| N | 71 _Subject.f_ | 71 _Subject.f_ |
| Observations | 3926 | 3926 |
| Marginal R^2^ / Conditional R^2^ | 0.348 / 0.613 | 0.339 / 0.600 |
| Deviance | 1859.320 | 1896.650 |
| AIC | 1922.733 | 1958.790 |
| ** p<0.05   ** p<0.01   *** p<0.001* | | |

**C. TTPV (log-transformed)**

|  | **Re1** | **Re2** |
| --- | --- | --- |
| *Predictors* | *Estimates* | *Estimates* |
| (Intercept) | 0.28 ^***^ (0.25 – 0.32) | 0.28 ^***^ (0.25 – 0.32) |
| TargetDist.c | 1.13 ^***^ (1.10 – 1.15) | 1.13 ^***^ (1.11 – 1.14) |
| Age.c | 0.74 ^***^ (0.66 – 0.84) | 0.75 ^***^ (0.66 – 0.84) |
| ASD.f [ASD] | 1.20  (1.00 – 1.44) | 1.19  (1.00 – 1.43) |
| TargetDist.c * Age.c | 0.99  (0.97 – 1.01) | 0.99  (0.98 – 1.00) |
| Age.c * ASD.f [ASD] | 1.21  (0.99 – 1.48) | 1.20  (0.98 – 1.47) |
| TargetDist.c * ASD.f [ASD] | 1.00  (0.96 – 1.03) | 1.01  (0.98 – 1.03) |
| **Random Effects** | | |
| σ^2^ | 0.21 | 0.22 |
| τ_00_ | 0.14 _Subject.f_ | 0.14 _Subject.f_ |
| τ_11_ | 0.00 _Subject.f.TargetDist.c_ |  |
| ρ_01_ | -0.10 _Subject.f_ |  |
| ICC | 0.40 | 0.38 |
| N | 71 _Subject.f_ | 71 _Subject.f_ |
| Observations | 3926 | 3926 |
| Marginal R^2^ / Conditional R^2^ | 0.199 / 0.516 | 0.198 / 0.504 |
| Deviance | 5397.585 | 5426.409 |
| AIC | 5454.910 | 5482.472 |
| ** p<0.05   ** p<0.01   *** p<0.001* | | |

**D. PV1 (mm/s)**

|  | **Re1** | **Re2** |
| --- | --- | --- |
| *Predictors* | *Estimates* | *Estimates* |
| (Intercept) | 116.95 ^***^ (107.05 – 126.85) | 117.76 ^***^ (107.60 – 127.91) |
| TargetDist.c | 14.76 ^***^ (12.39 – 17.13) | 15.46 ^***^ (14.13 – 16.79) |
| Age.c | 28.28 ^***^ (18.12 – 38.45) | 31.58 ^***^ (20.78 – 42.38) |
| ASD.f [ASD] | -16.72 ^*^ (-32.47 – -0.96) | -18.81 ^*^ (-35.01 – -2.61) |
| TargetDist.c * Age.c | 4.95 ^***^ (2.83 – 7.08) | 5.00 ^***^ (3.74 – 6.26) |
| Age.c * ASD.f [ASD] | -10.55  (-26.46 – 5.37) | -20.10 ^*^ (-38.14 – -2.06) |
| TargetDist.c * ASD.f [ASD] | 0.03  (-3.87 – 3.93) | 0.33  (-2.02 – 2.69) |
| **Random Effects** | | |
| σ^2^ | 2169.93 | 2239.36 |
| τ_00_ | 1005.15 _Subject.f_ | 1056.43 _Subject.f_ |
| τ_11_ | 39.86 _Subject.f.TargetDist.c_ |  |
| ρ_01_ | 0.63 _Subject.f_ |  |
| ICC | 0.35 | 0.32 |
| N | 71 _Subject.f_ | 71 _Subject.f_ |
| Observations | 3926 | 3926 |
| Marginal R^2^ / Conditional R^2^ | 0.251 / 0.512 | 0.276 / 0.508 |
| Deviance | 41573.078 | 41643.942 |
| AIC | 41567.505 | 41636.414 |
| ** p<0.05   ** p<0.01   *** p<0.001* | | |

**E. MU**

|  | **Re1** | **Re2** |
| --- | --- | --- |
| *Predictors* | *Incidence Rate Ratios* | *Incidence Rate Ratios* |
| (Intercept) | 0.85 ^*^ (0.72 – 1.00) | 0.86  (0.74 – 1.00) |
| TargetDist.c | 1.27 ^***^ (1.22 – 1.32) | 1.25 ^***^ (1.21 – 1.29) |
| Age.c | 0.60 ^***^ (0.51 – 0.71) | 0.60 ^***^ (0.51 – 0.71) |
| ASD.f [ASD] | 1.44 ^**^ (1.11 – 1.86) | 1.42 ^**^ (1.12 – 1.80) |
| TargetDist.c * Age.c | 1.02  (0.98 – 1.05) | 1.01  (0.98 – 1.04) |
| Age.c * ASD.f [ASD] | 1.32 ^*^ (1.04 – 1.68) | 1.34 ^*^ (1.03 – 1.74) |
| TargetDist.c * ASD.f [ASD] | 0.93 ^*^ (0.88 – 0.99) | 0.94 ^*^ (0.89 – 0.99) |
| **Random Effects** | | |
| σ^2^ | 0.18 | 0.39 |
| τ_00_ | 0.24 _Subject.f_ | 0.21 _Subject.f_ |
| τ_11_ | 0.00 _Subject.f.TargetDist.c_ |  |
| ρ_01_ | -0.91 _Subject.f_ |  |
| ICC | 0.56 | 0.35 |
| N | 71 _Subject.f_ | 71 _Subject.f_ |
| Observations | 3926 | 3926 |
| Marginal R^2^ / Conditional R^2^ | 0.389 / 0.734 | 0.301 / 0.544 |
| AIC | 7999.112 | 8006.590 |
| ** p<0.05   ** p<0.01   *** p<0.001* | | |

**F. %Dec (%)**

|  | **Re1** | **Re2** |
| --- | --- | --- |
| *Predictors* | *Estimates* | *Estimates* |
| (Intercept) | 55.32 ^***^ (53.16 – 57.47) | 55.31 ^***^ (53.22 – 57.39) |
| TargetDist.c | -0.57  (-1.25 – 0.10) | -0.53 ^*^ (-0.98 – -0.08) |
| Age.c | 0.70  (-1.57 – 2.97) | 0.50  (-1.73 – 2.72) |
| ASD.f [ASD] | -1.36  (-4.82 – 2.11) | -1.18  (-4.53 – 2.18) |
| TargetDist.c * Age.c | 0.20  (-0.42 – 0.82) | 0.17  (-0.26 – 0.60) |
| Age.c * ASD.f [ASD] | 1.42  (-2.33 – 5.18) | 1.97  (-1.81 – 5.75) |
| TargetDist.c * ASD.f [ASD] | -0.53  (-1.67 – 0.61) | -0.75  (-1.55 – 0.05) |
| **Random Effects** | | |
| σ^2^ | 254.90 | 259.39 |
| τ_00_ | 44.48 _Subject.f_ | 41.24 _Subject.f_ |
| τ_11_ | 2.57 _Subject.f.TargetDist.c_ |  |
| ρ_01_ | -0.37 _Subject.f_ |  |
| ICC | 0.16 | 0.14 |
| N | 71 _Subject.f_ | 71 _Subject.f_ |
| Observations | 3926 | 3926 |
| Marginal R^2^ / Conditional R^2^ | 0.010 / 0.165 | 0.011 / 0.147 |
| Deviance | 33089.096 | 33110.513 |
| AIC | 33102.487 | 33122.037 |
| ** p<0.05   ** p<0.01   *** p<0.001* | | |

**G. MU-APV**

|  | **Re1** | **Re2** |
| --- | --- | --- |
| *Predictors* | *Incidence Rate Ratios* | *Incidence Rate Ratios* |
| (Intercept) | 0.29 ^***^ (0.24 – 0.35) | 0.30 ^***^ (0.25 – 0.35) |
| TargetDist.c | 1.33 ^***^ (1.26 – 1.41) | 1.31 ^***^ (1.25 – 1.37) |
| Age.c | 0.60 ^***^ (0.50 – 0.73) | 0.61 ^***^ (0.50 – 0.73) |
| ASD.f [ASD] | 1.36 ^*^ (1.01 – 1.83) | 1.35 ^*^ (1.03 – 1.77) |
| TargetDist.c * Age.c | 1.00  (0.96 – 1.05) | 1.00  (0.96 – 1.04) |
| Age.c * ASD.f [ASD] | 1.36 ^*^ (1.02 – 1.80) | 1.36 ^*^ (1.01 – 1.83) |
| TargetDist.c * ASD.f [ASD] | 0.91 ^*^ (0.84 – 0.99) | 0.91 ^*^ (0.85 – 0.99) |
| **Random Effects** | | |
| σ^2^ | 1.17 | 1.25 |
| τ_00_ | 0.30 _Subject.f_ | 0.24 _Subject.f_ |
| τ_11_ | 0.01 _Subject.f.TargetDist.c_ |  |
| ρ_01_ | -0.86 _Subject.f_ |  |
| ICC | 0.20 | 0.16 |
| N | 71 _Subject.f_ | 71 _Subject.f_ |
| Observations | 3926 | 3926 |
| Marginal R^2^ / Conditional R^2^ | 0.168 / 0.332 | 0.156 / 0.294 |
| Deviance | 6083.554 | 6092.208 |
| AIC | 6103.554 | 6108.208 |
| ** p<0.05   ** p<0.01   *** p<0.001* | | |

**H. PV1-b**

|  | **Re1** | **Re2** |
| --- | --- | --- |
| *Predictors* | *Odds Ratios* | *Odds Ratios* |
| (Intercept) | 4.62 ^***^ (3.52 – 6.06) | 4.49 ^***^ (3.47 – 5.79) |
| TargetDist.c | 0.74 ^***^ (0.68 – 0.82) | 0.76 ^***^ (0.70 – 0.82) |
| Age.c | 2.18 ^***^ (1.65 – 2.88) | 2.15 ^***^ (1.64 – 2.83) |
| ASD.f [ASD] | 0.52 ^**^ (0.34 – 0.79) | 0.53 ^**^ (0.36 – 0.79) |
| TargetDist.c * Age.c | 0.99  (0.91 – 1.08) | 1.00  (0.93 – 1.07) |
| Age.c * ASD.f [ASD] | 0.68  (0.44 – 1.07) | 0.69  (0.44 – 1.07) |
| TargetDist.c * ASD.f [ASD] | 1.02  (0.88 – 1.18) | 1.00  (0.89 – 1.12) |
| **Random Effects** | | |
| σ^2^ | 3.29 | 3.29 |
| τ_00_ | 0.60 _Subject.f_ | 0.53 _Subject.f_ |
| τ_11_ | 0.03 _Subject.f.TargetDist.c_ |  |
| ρ_01_ | -0.54 _Subject.f_ |  |
| ICC | 0.16 | 0.14 |
| N | 71 _Subject.f_ | 71 _Subject.f_ |
| Observations | 3926 | 3926 |
| Marginal R^2^ / Conditional R^2^ | 0.138 / 0.273 | 0.135 / 0.255 |
| Deviance | 3943.365 | 3950.188 |
| AIC | 3963.365 | 3966.188 |
| ** p<0.05   ** p<0.01   *** p<0.001* | | |

**Supplemental Tables 5A – 5H. Model building (Fixed effects).
Models Fe1 – Fe4** for MT, PV, TTPV, and PV1, %Dec, MU-APV, PV1-b, and MU-APV

**A. MT (log-transformed)**

|  | **Fe1** | **Fe2** | **Fe3** | **Fe4** |
| --- | --- | --- | --- | --- |
| *Predictors* | *Estimates* | *Estimates* | *Estimates* | *Estimates* |
| (Intercept) | 0.68 ^***^ (0.61 – 0.76) | 0.68 ^***^ (0.61 – 0.76) | 0.70 ^***^ (0.63 – 0.77) | 0.71 ^***^ (0.63 – 0.79) |
| TargetDist.c | 1.11 ^***^ (1.10 – 1.13) | 1.11 ^***^ (1.10 – 1.13) | 1.10 ^***^ (1.09 – 1.12) | 1.10 ^***^ (1.09 – 1.12) |
| Age.c | 0.73 ^***^ (0.65 – 0.81) | 0.71 ^***^ (0.64 – 0.79) | 0.71 ^***^ (0.64 – 0.79) | 0.79 ^***^ (0.72 – 0.87) |
| ASD.f [ASD] | 1.19 ^*^ (1.01 – 1.42) | 1.20 ^*^ (1.01 – 1.42) | 1.13  (0.97 – 1.32) | 1.09  (0.93 – 1.29) |
| TargetDist.c * Age.c | 0.99  (0.98 – 1.01) |  |  |  |
| Age.c * ASD.f [ASD] | 1.33 ^**^ (1.12 – 1.59) | 1.34 ^**^ (1.13 – 1.60) | 1.33 ^**^ (1.12 – 1.59) |  |
| TargetDist.c * ASD.f [ASD] | 0.98  (0.96 – 1.00) | 0.98  (0.96 – 1.00) |  |  |
| **Random Effects** | | | | |
| σ^2^ | 0.08 | 0.08 | 0.08 | 0.08 |
| τ_00_ | 0.12 _Subject.f_ | 0.12 _Subject.f_ | 0.12 _Subject.f_ | 0.13 _Subject.f_ |
| τ_11_ | 0.00 _Subject.f.TargetDist.c_ | 0.00 _Subject.f.TargetDist.c_ | 0.00 _Subject.f.TargetDist.c_ | 0.00 _Subject.f.TargetDist.c_ |
| ρ_01_ | -0.49 _Subject.f_ | -0.49 _Subject.f_ | -0.49 _Subject.f_ | -0.38 _Subject.f_ |
| ICC | 0.60 | 0.60 | 0.60 | 0.62 |
| N | 71 _Subject.f_ | 71 _Subject.f_ | 71 _Subject.f_ | 71 _Subject.f_ |
| Observations | 3926 | 3926 | 3926 | 3926 |
| Marginal R^2^ / Conditional R^2^ | 0.298 / 0.719 | 0.315 / 0.726 | 0.303 / 0.722 | 0.229 / 0.706 |
| AIC | 1562.360 | 1561.580 | 1562.313 | 1569.739 |
| ** p<0.05   ** p<0.01   *** p<0.001* | | | | |

**B. PV (log-transformed)**

|  | **Fe1** | **Fe2** | **Fe3** | **Fe4** |
| --- | --- | --- | --- | --- |
| *Predictors* | *Estimates* | *Estimates* | *Estimates* | *Estimates* |
| (Intercept) | 114.75 ^***^ (106.67 – 123.45) | 114.91 ^***^ (106.83 – 123.61) | 114.12 ^***^ (106.19 – 122.63) | 113.18 ^***^ (105.17 – 121.79) |
| TargetDist.c | 1.16 ^***^ (1.15 – 1.18) | 1.16 ^***^ (1.14 – 1.18) | 1.17 ^***^ (1.15 – 1.18) | 1.17 ^***^ (1.15 – 1.18) |
| Age.c | 1.24 ^***^ (1.15 – 1.34) | 1.25 ^***^ (1.16 – 1.35) | 1.25 ^***^ (1.16 – 1.35) | 1.20 ^***^ (1.12 – 1.27) |
| ASD.f [ASD] | 0.90  (0.80 – 1.01) | 0.90  (0.80 – 1.01) | 0.92  (0.82 – 1.03) | 0.94  (0.84 – 1.05) |
| TargetDist.c * Age.c | 1.00  (0.99 – 1.02) |  |  |  |
| Age.c * ASD.f [ASD] | 0.88 ^*^ (0.78 – 1.00) | 0.88 ^*^ (0.77 – 0.99) | 0.88 ^*^ (0.78 – 1.00) |  |
| TargetDist.c * ASD.f [ASD] | 1.01  (0.99 – 1.04) | 1.01  (0.99 – 1.04) |  |  |
| **Random Effects** | | | | |
| σ^2^ | 0.09 | 0.09 | 0.09 | 0.09 |
| τ_00_ | 0.06 _Subject.f_ | 0.06 _Subject.f_ | 0.06 _Subject.f_ | 0.06 _Subject.f_ |
| τ_11_ | 0.00 _Subject.f.TargetDist.c_ | 0.00 _Subject.f.TargetDist.c_ | 0.00 _Subject.f.TargetDist.c_ | 0.00 _Subject.f.TargetDist.c_ |
| ρ_01_ | -0.33 _Subject.f_ | -0.34 _Subject.f_ | -0.33 _Subject.f_ | -0.30 _Subject.f_ |
| ICC | 0.39 | 0.39 | 0.39 | 0.40 |
| N | 71 _Subject.f_ | 71 _Subject.f_ | 71 _Subject.f_ | 71 _Subject.f_ |
| Observations | 3926 | 3926 | 3926 | 3926 |
| Marginal R^2^ / Conditional R^2^ | 0.353 / 0.607 | 0.357 / 0.609 | 0.355 / 0.608 | 0.331 / 0.601 |
| AIC | 1881.129 | 1879.697 | 1879.008 | 1880.927 |
| ** p<0.05   ** p<0.01   *** p<0.001* | | | | |

**C. TTPV (log-transformed)**

|  | **Fe1** | **Fe2** | **Fe3** | **Fe4** |
| --- | --- | --- | --- | --- |
| *Predictors* | *Estimates* | *Estimates* | *Estimates* | *Estimates* |
| (Intercept) | 0.28 ^***^ (0.25 – 0.31) | 0.28 ^***^ (0.25 – 0.31) | 0.28 ^***^ (0.25 – 0.31) | 0.29 ^***^ (0.25 – 0.32) |
| TargetDist.c | 1.13 ^***^ (1.10 – 1.15) | 1.13 ^***^ (1.11 – 1.15) | 1.13 ^***^ (1.11 – 1.15) | 1.13 ^***^ (1.11 – 1.15) |
| Age.c | 0.74 ^***^ (0.66 – 0.84) | 0.74 ^***^ (0.66 – 0.83) | 0.74 ^***^ (0.66 – 0.83) | 0.79 ^***^ (0.72 – 0.87) |
| ASD.f [ASD] | 1.20 ^*^ (1.01 – 1.44) | 1.20 ^*^ (1.01 – 1.44) | 1.20 ^*^ (1.01 – 1.43) | 1.17  (0.98 – 1.39) |
| TargetDist.c * Age.c | 0.99  (0.97 – 1.01) |  |  |  |
| Age.c * ASD.f [ASD] | 1.21  (0.99 – 1.47) | 1.21  (0.99 – 1.47) | 1.21  (0.99 – 1.47) |  |
| TargetDist.c * ASD.f [ASD] | 1.00  (0.96 – 1.03) | 1.00  (0.96 – 1.03) |  |  |
| **Random Effects** | | | | |
| σ^2^ | 0.21 | 0.21 | 0.21 | 0.21 |
| τ_00_ | 0.13 _Subject.f_ | 0.13 _Subject.f_ | 0.13 _Subject.f_ | 0.13 _Subject.f_ |
| τ_11_ | 0.00 _Subject.f.TargetDist.c_ | 0.00 _Subject.f.TargetDist.c_ | 0.00 _Subject.f.TargetDist.c_ | 0.00 _Subject.f.TargetDist.c_ |
| ρ_01_ | -0.10 _Subject.f_ | -0.11 _Subject.f_ | -0.11 _Subject.f_ | -0.01 _Subject.f_ |
| ICC | 0.38 | 0.38 | 0.38 | 0.39 |
| N | 71 _Subject.f_ | 71 _Subject.f_ | 71 _Subject.f_ | 71 _Subject.f_ |
| Observations | 3926 | 3926 | 3926 | 3926 |
| Marginal R^2^ / Conditional R^2^ | 0.202 / 0.507 | 0.205 / 0.508 | 0.204 / 0.508 | 0.174 / 0.498 |
| AIC | 5419.400 | 5418.315 | 5416.337 | 5417.542 |
| ** p<0.05   ** p<0.01   *** p<0.001* | | | | |

**D. PV1 (mm/s).**Fe3 and Fe4 failed to converge

|  | **Fe1** | **Fe2** | **Fe3** | **Fe4** |
| --- | --- | --- | --- | --- |
| *Predictors* | *Estimates* | *Estimates* | *Estimates* | *Estimates* |
| (Intercept) | 116.98 ^***^ (107.35 – 126.61) | 115.19 ^***^ (105.31 – 125.07) | 117.00 ^*^ (107.79 – 126.22) | 116.37 ^***^ (107.01 – 125.73) |
| TargetDist.c | 14.76 ^***^ (12.44 – 17.07) | 14.06 ^***^ (11.40 – 16.73) | 14.77 ^***^ (12.92 – 16.62) | 14.74 ^***^ (12.89 – 16.60) |
| Age.c | 28.28 ^***^ (18.40 – 38.17) | 19.16 ^***^ (10.06 – 28.27) | 28.28  (18.40 – 38.17) | 24.52  (16.09 – 32.95) |
| ASD.f [ASD] | -16.73 ^*^ (-32.06 – -1.40) | -16.12 ^*^ (-31.91 – -0.34) | -16.79 ^*^ (-30.51 – -3.07) | -15.37 ^*^ (-29.08 – -1.65) |
| TargetDist.c * Age.c | 4.97 ^***^ (2.89 – 7.04) |  | 4.97  (2.89 – 7.04) | 4.89  (2.82 – 6.97) |
| Age.c * ASD.f [ASD] | -10.62  (-26.11 – 4.86) | -8.80  (-24.42 – 6.81) | -10.62  (-26.09 – 4.85) |  |
| TargetDist.c * ASD.f [ASD] | 0.03  (-3.78 – 3.84) | 0.06  (-4.31 – 4.42) |  |  |
| **Random Effects** | | | | |
| σ^2^ | 2169.82 | 2169.21 | 2169.80 | 2169.53 |
| τ_00_ | 948.55 _Subject.f_ | 1010.09 _Subject.f_ | 949.41 _Subject.f_ | 1000.77 _Subject.f_ |
| τ_11_ | 37.15 _Subject.f.TargetDist.c_ | 56.26 _Subject.f.TargetDist.c_ | 37.15 _Subject.f.TargetDist.c_ | 37.63 _Subject.f.TargetDist.c_ |
| ρ_01_ | 0.65 _Subject.f_ | 0.64 _Subject.f_ | 0.65 _Subject.f_ | 0.67 _Subject.f_ |
| ICC | 0.34 | 0.36 | 0.34 | 0.35 |
| N | 71 _Subject.f_ | 71 _Subject.f_ | 71 _Subject.f_ | 71 _Subject.f_ |
| Observations | 3926 | 3926 | 3926 | 3926 |
| Marginal R^2^/ Conditional R^2^ | 0.254 / 0.505 | 0.167 / 0.466 | 0.254 / 0.505 | 0.239 / 0.503 |
| AIC | 41594.902 | 41611.862 | 41592.903 | 41592.591 |
| ** p<0.05   ** p<0.01   *** p<0.001* | | | | |

**E. MU**

|  | **Fe1** | **Fe2** | **Fe3** | **Fe4** |
| --- | --- | --- | --- | --- |
| *Predictors* | *Incidence Rate Ratios* | *Incidence Rate Ratios* | *Incidence Rate Ratios* | *Incidence Rate Ratios* |
| (Intercept) | 0.83 ^*^ (0.71 – 0.98) | 0.84 ^*^ (0.72 – 0.99) | 0.90  (0.77 – 1.04) | 0.86  (0.73 – 1.01) |
| TargetDist.c | 1.27 ^***^ (1.22 – 1.32) | 1.26 ^***^ (1.21 – 1.31) | 1.23 ^***^ (1.19 – 1.27) | 1.26 ^***^ (1.21 – 1.31) |
| Age.c | 0.60 ^***^ (0.51 – 0.70) | 0.63 ^***^ (0.54 – 0.72) | 0.62 ^***^ (0.54 – 0.72) | 0.69 ^***^ (0.62 – 0.78) |
| ASD.f [ASD] | 1.44 ^**^ (1.12 – 1.84) | 1.43 ^**^ (1.11 – 1.83) | 1.23  (0.99 – 1.52) | 1.36 ^*^ (1.06 – 1.75) |
| TargetDist.c * Age.c | 1.02  (0.99 – 1.05) |  |  |  |
| Age.c * ASD.f [ASD] | 1.32 ^*^ (1.05 – 1.67) | 1.32 ^*^ (1.05 – 1.67) | 1.33 ^*^ (1.06 – 1.68) |  |
| TargetDist.c * ASD.f [ASD] | 0.93 ^*^ (0.88 – 0.99) | 0.94 ^*^ (0.88 – 0.99) |  | 0.93 ^*^ (0.88 – 0.99) |
| **Random Effects** | | | | |
| σ^2^ | 0.19 | 0.19 | 0.19 | 0.19 |
| τ_00_ | 0.23 _Subject.f_ | 0.23 _Subject.f_ | 0.23 _Subject.f_ | 0.24 _Subject.f_ |
| τ_11_ | 0.00 _Subject.f.TargetDist.c_ | 0.00 _Subject.f.TargetDist.c_ | 0.00 _Subject.f.TargetDist.c_ | 0.00 _Subject.f.TargetDist.c_ |
| ρ_01_ | -0.98 _Subject.f_ | -0.97 _Subject.f_ | -0.94 _Subject.f_ | -0.96 _Subject.f_ |
| ICC | 0.54 | 0.53 | 0.54 | 0.55 |
| N | 71 _Subject.f_ | 71 _Subject.f_ | 71 _Subject.f_ | 71 _Subject.f_ |
| Observations | 3926 | 3926 | 3926 | 3926 |
| Marginal R^2^ / Conditional R^2^ | 0.397 / 0.720 | 0.372 / 0.708 | 0.351 / 0.700 | 0.332 / 0.699 |
| AIC | 7967.920 | 7967.121 | 7969.720 | 7970.555 |
| ** p<0.05   ** p<0.01   *** p<0.001* | | | | |

**F. %Dec (%)**

|  | **Fe1** | **Fe2** | **Fe3** | **Fe4** |
| --- | --- | --- | --- | --- |
| *Predictors* | *Estimates* | *Estimates* | *Estimates* | *Estimates* |
| (Intercept) | 55.32 ^***^ (53.23 – 57.41) | 55.36 ^***^ (53.26 – 57.45) | 55.54 ^***^ (53.48 – 57.60) | 55.62 ^***^ (53.56 – 57.68) |
| TargetDist.c | -0.57  (-1.23 – 0.09) | -0.59  (-1.25 – 0.07) | -0.78 ^**^ (-1.32 – -0.24) | -0.78 ^**^ (-1.32 – -0.24) |
| Age.c | 0.69  (-1.51 – 2.90) | 0.88  (-1.25 – 3.01) | 0.92  (-1.21 – 3.04) | 1.37  (-0.36 – 3.10) |
| ASD.f [ASD] | -1.34  (-4.71 – 2.03) | -1.36  (-4.74 – 2.01) | -1.87  (-5.07 – 1.33) | -2.08  (-5.25 – 1.09) |
| TargetDist.c * Age.c | 0.19  (-0.41 – 0.80) |  |  |  |
| Age.c * ASD.f [ASD] | 1.41  (-2.24 – 5.06) | 1.36  (-2.28 – 5.01) | 1.31  (-2.33 – 4.95) |  |
| TargetDist.c * ASD.f [ASD] | -0.54  (-1.65 – 0.57) | -0.54  (-1.65 – 0.58) |  |  |
| **Random Effects** | | | | |
| σ^2^ | 254.91 | 254.93 | 254.88 | 254.90 |
| τ_00_ | 41.62 _Subject.f_ | 41.75 _Subject.f_ | 41.88 _Subject.f_ | 42.53 _Subject.f_ |
| τ_11_ | 2.33 _Subject.f.TargetDist.c_ | 2.34 _Subject.f.TargetDist.c_ | 2.47 _Subject.f.TargetDist.c_ | 2.47 _Subject.f.TargetDist.c_ |
| ρ_01_ | -0.38 _Subject.f_ | -0.39 _Subject.f_ | -0.39 _Subject.f_ | -0.42 _Subject.f_ |
| ICC | 0.15 | 0.15 | 0.15 | 0.15 |
| N | 71 _Subject.f_ | 71 _Subject.f_ | 71 _Subject.f_ | 71 _Subject.f_ |
| Observations | 3926 | 3926 | 3926 | 3926 |
| Marginal R^2^/ Conditional R^2^ | 0.010 / 0.156 | 0.011 / 0.157 | 0.012 / 0.158 | 0.012 / 0.160 |
| AIC | 33110.909 | 33109.305 | 33108.171 | 33106.646 |
| ** p<0.05   ** p<0.01   *** p<0.001* | | | | |

**G. MU-APV**

|  | **Fe1** | **Fe2** | **Fe3** | **Fe4** |
| --- | --- | --- | --- | --- |
| *Predictors* | *Incidence Rate Ratios* | *Incidence Rate Ratios* | *Incidence Rate Ratios* | *Incidence Rate Ratios* |
| (Intercept) | 0.29 ^***^ (0.24 – 0.35) | 0.29 ^***^ (0.24 – 0.35) | 0.31 ^***^ (0.26 – 0.37) | 0.30 ^***^ (0.25 – 0.36) |
| TargetDist.c | 1.33 ^***^ (1.26 – 1.41) | 1.33 ^***^ (1.26 – 1.41) | 1.29 ^***^ (1.23 – 1.35) | 1.33 ^***^ (1.26 – 1.41) |
| Age.c | 0.60 ^***^ (0.50 – 0.73) | 0.61 ^***^ (0.51 – 0.72) | 0.61 ^***^ (0.51 – 0.72) | 0.67 ^***^ (0.59 – 0.77) |
| ASD.f [ASD] | 1.36 ^*^ (1.01 – 1.83) | 1.36 ^*^ (1.01 – 1.83) | 1.15  (0.88 – 1.49) | 1.29  (0.96 – 1.74) |
| TargetDist.c * Age.c | 1.00  (0.96 – 1.05) |  |  |  |
| Age.c * ASD.f [ASD] | 1.36 ^*^ (1.02 – 1.80) | 1.35 ^*^ (1.02 – 1.80) | 1.36 ^*^ (1.02 – 1.81) |  |
| TargetDist.c * ASD.f [ASD] | 0.91 ^*^ (0.84 – 0.99) | 0.91 ^*^ (0.84 – 0.99) |  | 0.91 ^*^ (0.83 – 0.99) |
| **Random Effects** | | | | |
| σ^2^ | 1.17 | 1.17 | 1.17 | 1.17 |
| τ_00_ | 0.30 _Subject.f_ | 0.30 _Subject.f_ | 0.30 _Subject.f_ | 0.31 _Subject.f_ |
| τ_11_ | 0.01 _Subject.f.TargetDist.c_ | 0.01 _Subject.f.TargetDist.c_ | 0.01 _Subject.f.TargetDist.c_ | 0.01 _Subject.f.TargetDist.c_ |
| ρ_01_ | -0.86 _Subject.f_ | -0.86 _Subject.f_ | -0.79 _Subject.f_ | -0.82 _Subject.f_ |
| ICC | 0.20 | 0.20 | 0.20 | 0.21 |
| N | 71 _Subject.f_ | 71 _Subject.f_ | 71 _Subject.f_ | 71 _Subject.f_ |
| Observations | 3926 | 3926 | 3926 | 3926 |
| Marginal R^2^/ Conditional R^2^ | 0.168 / 0.332 | 0.166 / 0.331 | 0.155 / 0.326 | 0.149 / 0.324 |
| AIC | 6103.554 | 6101.565 | 6104.143 | 6103.817 |
| ** p<0.05   ** p<0.01   *** p<0.001* | | | | |

**H. PV1-b**

|  | **Fe1** | **Fe2** | **Fe3** | **Fe4** |
| --- | --- | --- | --- | --- |
| *Predictors* | *Odds Ratios* | *Odds Ratios* | *Odds Ratios* | *Odds Ratios* |
| (Intercept) | 4.62 ^***^ (3.52 – 6.06) | 4.61 ^***^ (3.52 – 6.02) | 4.57 ^***^ (3.52 – 5.92) | 4.45 ^***^ (3.43 – 5.78) |
| TargetDist.c | 0.74 ^***^ (0.68 – 0.82) | 0.75 ^***^ (0.68 – 0.82) | 0.75 ^***^ (0.70 – 0.81) | 0.75 ^***^ (0.70 – 0.81) |
| Age.c | 2.18 ^***^ (1.65 – 2.88) | 2.16 ^***^ (1.65 – 2.82) | 2.16 ^***^ (1.65 – 2.82) | 1.89 ^***^ (1.52 – 2.35) |
| ASD.f [ASD] | 0.52 ^**^ (0.34 – 0.79) | 0.52 ^**^ (0.34 – 0.79) | 0.53 ^**^ (0.36 – 0.78) | 0.56 ^**^ (0.38 – 0.82) |
| TargetDist.c * Age.c | 0.99  (0.91 – 1.08) |  |  |  |
| Age.c * ASD.f [ASD] | 0.68  (0.44 – 1.07) | 0.68  (0.44 – 1.07) | 0.68  (0.44 – 1.07) |  |
| TargetDist.c * ASD.f [ASD] | 1.02  (0.88 – 1.18) | 1.02  (0.88 – 1.18) |  |  |
| **Random Effects** | | | | |
| σ^2^ | 3.29 | 3.29 | 3.29 | 3.29 |
| τ_00_ | 0.60 _Subject.f_ | 0.60 _Subject.f_ | 0.60 _Subject.f_ | 0.62 _Subject.f_ |
| τ_11_ | 0.03 _Subject.f.TargetDist.c_ | 0.03 _Subject.f.TargetDist.c_ | 0.03 _Subject.f.TargetDist.c_ | 0.03 _Subject.f.TargetDist.c_ |
| ρ_01_ | -0.54 _Subject.f_ | -0.53 _Subject.f_ | -0.53 _Subject.f_ | -0.52 _Subject.f_ |
| ICC | 0.16 | 0.16 | 0.16 | 0.16 |
| N | 71 _Subject.f_ | 71 _Subject.f_ | 71 _Subject.f_ | 71 _Subject.f_ |
| Observations | 3926 | 3926 | 3926 | 3926 |
| Marginal R^2^/ Conditional R^2^ | 0.138 / 0.273 | 0.136 / 0.271 | 0.135 / 0.270 | 0.123 / 0.263 |
| AIC | 3963.365 | 3961.406 | 3959.460 | 3960.211 |
| ** p<0.05   ** p<0.01   *** p<0.001* | | | | |
